# Regional suicide prevention planning: a dynamic simulation modelling analysis

**DOI:** 10.1192/bjo.2021.989

**Published:** 2021-08-31

**Authors:** Adam Skinner, Jo-An Occhipinti, Yun Ju Christine Song, Ian B. Hickie

**Affiliations:** Brain and Mind Centre, Faculty of Medicine and Health, University of Sydney, Australia; Brain and Mind Centre, Faculty of Medicine and Health, University of Sydney, Australia; and Computer Simulation and Advanced Research Technologies, Australia; Brain and Mind Centre, Faculty of Medicine and Health, University of Sydney, Australia; Brain and Mind Centre, Faculty of Medicine and Health, University of Sydney, Australia

**Keywords:** Australia, computer simulation, mental health services, suicide, system dynamics

## Abstract

**Background:**

Regional planning may help to ensure that the specific measures implemented as part of a national suicide prevention strategy are aligned with the varying needs of local services and communities; however, there are concerns that the reliability of local programme development may be limited in practice.

**Aims:**

The potential impacts of independent regional planning on the effectiveness of suicide prevention programmes in the Australian state of New South Wales were quantified using a system dynamics model of mental health services provision and suicidal behaviour in each of the state's ten Primary Health Network (PHN) catchments.

**Method:**

Reductions in projected suicide mortality over the period 2021–2031 were calculated for scenarios in which combinations of four and five suicide prevention and mental health services interventions (selected from 13 possible interventions) are implemented separately in each PHN catchment. State-level impacts were estimated by summing reductions in projected suicide mortality for each intervention combination across PHN catchments.

**Results:**

The most effective state-level combinations of four and five interventions prevent, respectively, 20.3% and 22.9% of 10 312 suicides projected under a business-as-usual scenario (i.e. no new policies or programmes, constant services capacity growth). Projected numbers of suicides under the optimal intervention scenarios for each PHN are up to 6% lower than corresponding numbers of suicides projected for the optimal state-level intervention combinations.

**Conclusions:**

Regional suicide prevention planning may contribute to significant reductions in suicide mortality where local health authorities are provided with the necessary resources and tools to support reliable, evidence-based decision-making.

Preventing suicide is a global public health priority. More than 800 000 people are estimated to have died by suicide in 2016, including nearly 137 000 children and young adults aged 10–24 years.^1^ Among people aged 15–29 years, suicide is the second leading cause of mortality worldwide, accounting for around 8% of deaths in this age group every year.^2^ The World Health Organization proposed in its Global Mental Health Action Plan, 2013–2020 that national governments should develop and implement comprehensive suicide prevention strategies with the collective goal of achieving a 10% decrease in the global suicide rate by 2020.^3^ As of November 2018, 38 countries had adopted a national strategy developed specifically to prevent suicide and intentional self-harm.^4^ Typically, these strategies incorporate a broad range of objectives: restricting access to commonly-used means of suicide, increasing the availability of mental health services for people at risk of suicidal behaviour, providing improved care to people who have self-harmed, promoting responsible reporting of suicide by the media, increasing public awareness of suicide and mental disorders and improving suicide surveillance systems.^4^

National suicide prevention strategies are often implemented locally via (semi-)independent subnational health authorities to accommodate potentially substantial regional variation in the incidence and underlying causes of suicide and intentional self-harm.^4^ Multiple ecological studies have revealed significant heterogeneity in local suicide rates that may be ascribed (at least partially) to geographical variation in social and economic risk-modifying factors, including alcohol consumption, population density, unemployment, poverty and deprivation, and interpersonal violence.^5–10^ Partial (non-uniform) remodelling of mental healthcare provision and the dependence of policy intervention effects on local context (catchment population size, case mix and other factors affecting demand for services) may also contribute significantly to regional variation in suicide mortality.^6,11,12^ Nevertheless, while regional planning may help to ensure that the specific programmes and policies implemented as part of a national suicide prevention strategy are aligned with the varying needs of local services and communities, concerns that the reliability of regional decision-making may be limited in practice have prompted a shift toward more centralised suicide prevention planning in some countries.^13^

## Objectives

This paper presents an analysis of the potential impact of independent regional planning on the effectiveness of suicide prevention programmes, focusing on the Australian state of New South Wales (NSW). Using a system dynamics model of mental health services provision and suicidal behaviour in each of the state's ten Primary Health Network (PHN) catchments, we ran a set of simulation analyses designed to address three principal study aims: first, to determine the extent to which significant regional variation in population and health system characteristics (e.g. prevalence of mental disorders, mental health services accessibility and rates of increase in services capacity) modifies the effects of individual suicide prevention measures on local suicide rates; second, to quantify the potential impact of optimal regional planning (i.e. relative to state-level planning) on the effectiveness of suicide prevention programmes comprising multiple interventions implemented in parallel; and third, to assess the capacity of suboptimal commissioning decisions to limit (or reverse) any potential benefit of local programme development.

## Method

### Context

NSW is the most populous state in Australia, with an estimated resident population of 8.16 million (as of 31 March 2020), comprising 31.8% of the total Australian population.^14^ Government-funded suicide prevention services provided in the state are commissioned locally via ten PHNs, each of which supports a geographically defined population of between approximately 243 000 and 1.57 million residents (based on 2016 population estimates; see Supplementary Table 1 available at https://doi.org/10.1192/bjo.2021.989). Population density varies substantially among PHN catchments, from 2512 people per square kilometre for Central and Eastern Sydney PHN to <1 person per square kilometre for Western NSW PHN. The distribution of socioeconomic disadvantage across catchments is also highly uneven: 44.2% of the South Western Sydney PHN catchment population resides in Level 2 Statistical Areas (SA2s) with Index of Relative Socio-Economic Disadvantage scores in the lowest quintile for the state,^15^ whereas the Northern Sydney PHN catchment contains no SA2s with an Index of Relative Socio-Economic Disadvantage score in the lowest quintile (i.e. all residents live in less disadvantaged SA2s). Mean yearly suicide rates for the period 2001–2017 range from 7.61 suicides per 10^5^ population for Western Sydney PHN to 12.18 suicides per 10^5^ population for North Coast PHN (Supplementary Table 1).

### Model structure, outputs and calibration

The system dynamics model used for the analyses presented here is based on a similar model recently developed for Hunter New England and Central Coast PHN using a participatory modelling approach that involved diverse stakeholders, including representatives from health and social policy agencies, local government, non-government organisations, primary care providers, emergency services, research institutions, community groups and people with lived experience of mental illness and intentional self-harm.^16^ The core model structure, replicated for each PHN, comprises a set of interconnected sub-models or sectors, including: (a) a population sector, capturing changes in catchment population size resulting from births, migration and mortality; (b) a psychological distress sector that models flows of people to and from states of low psychological distress (Kessler-10 score of 10–15) and moderate to very high psychological distress (Kessler-10 score of 16–50); (c) a mental health services sector, modelling the movement of psychologically distressed patients through a network of possible service pathways involving general practitioners, psychiatrists and allied mental healthcare providers, psychiatric in-patient care, community mental healthcare services and online services and (d) a suicidal behaviour sector that captures numbers of self-harm hospitalisations and suicides. Detailed descriptions of all model sectors are provided in the Supplementary Material. Parameter estimates and other numerical inputs were derived from published research or publicly available data (where possible), or were estimated via constrained optimisation (see below). Model construction and analysis were performed with Stella Architect version 1.9.4 (isee systems, Lebanon, NH, USA; see www.iseesystems.com).

All data used in this study are publicly available, so ethical approval was not required.

Primary model outputs include total (cumulative) numbers of self-harm hospitalisations and suicides (calculated for each PHN catchment and the state as a whole) and numbers of self-harm hospitalisations and suicides per year. The model also provides estimates of the prevalence of moderate to very high psychological distress and a range of services-related outcomes, including numbers of mental health-related emergency department presentations (total and per year), numbers of general practice and community-based specialised mental health services consultations, numbers of psychiatric and acute (general) hospital admissions, hospital and community-based services waiting times, and numbers of patients disengaging from services because of excessive waiting times or dissatisfaction with the care they receive. Outputs are calculated every 0.875 days (the numerical integration time step, *dt*, was set to one-eighth of a week; see Sterman,^17^ Appendix A) over a period of 20 years, starting from 1 January 2011, so that the impacts of suicide prevention and mental health services interventions were modelled from the time of implementation to the start of 2031 (simulations were run from 2011 to permit comparisons of model outputs with historical data; see Supplementary Material). The analyses presented here focus on projected total numbers of suicides for the state and each PHN catchment.

Parameter values that could not be derived directly from available data or published research were estimated via constrained optimisation, implemented in Stella Architect version 1.9.4, using historical time-series data on the prevalence of moderate to very high psychological distress, self-harm hospitalisation and suicide mortality rates, and rates of mental health services usage (numbers of mental health-related emergency department presentations, general practice and community-based mental health services consultations, and psychiatric and general hospital admissions per year). Powell's method^18^ was employed to obtain the set of (optimal) parameter values minimising the mean of the absolute differences between the observed time-series values and the corresponding model outputs, where each difference was expressed as a percentage of the observed value (i.e. the mean absolute per cent error was used as the objective function for the optimisation analyses).^17^

### Simulation experiments and sensitivity analyses

Reductions in projected suicide mortality were calculated for intervention scenarios in which one or more of 13 suicide prevention and mental health services interventions are implemented in a single PHN catchment. Details of all interventions are provided in Supplementary Table 2. Note that only the direct effects of each intervention are specified explicitly in the model; indirect effects are generated automatically by the model structure, so that the total effects (direct and indirect) of separate interventions implemented in parallel are not necessarily additive (i.e. interventions may have synergistic or antagonistic effects when combined). PHN-specific reductions in suicide mortality were calculated for the 10-year period from 1 January 2021 to the start of 2031 by subtracting total (cumulative) numbers of suicides projected under a given intervention scenario from the total number of suicides projected under a baseline scenario (corresponding to business as usual) in which existing suicide prevention policies and services remain in place and mental health services capacity continues to increase at current rates. State-level intervention effects were obtained by summing PHN-specific reductions in projected numbers of suicides across PHN catchments.

Potential variation in the effects of individual interventions among PHN catchments was assessed by comparing percentage reductions in total numbers of suicides for the same intervention implemented separately in each catchment (study aim 1). We quantified the impact of optimal regional planning on the effectiveness of multi-component suicide prevention programmes comprising four and five interventions (study aim 2), by calculating differences between the maximum reduction in projected numbers of suicides for each PHN catchment (i.e. given the best combination of interventions for the catchment) and PHN-specific reductions in projected numbers of suicides under the optimal state-level intervention scenario (the combination of interventions minimising state-level suicide mortality when implemented in all PHN catchments). Our choice of intervention set sizes (i.e. 4–5) reflects the fact that suicide prevention programmes are generally implemented within resource-constrained settings, where only a limited number of interventions can be supported. The capacity of suboptimal intervention selection to limit any potential benefit of regional planning was assessed for each PHN catchment by determining the number of intervention scenarios preventing fewer suicides than the optimal state-level scenario (study aim 3).

Sensitivity analyses were performed to assess the impact of uncertainty in estimates of the direct effects of the interventions on the simulation results. We used Latin hypercube sampling to draw 100 sets of values for selected model parameters determining the direct effects of the interventions on psychological distress, suicidal behaviour and the flow of patients through mental health services from a uniform joint distribution spanning +/−20% of the default values (see Supplementary Material). Differences in projected (total) numbers of suicides between the baseline and intervention scenarios were calculated for each set of parameter values and summarised using simple descriptive statistics.

## Results

**Fig. 1:**
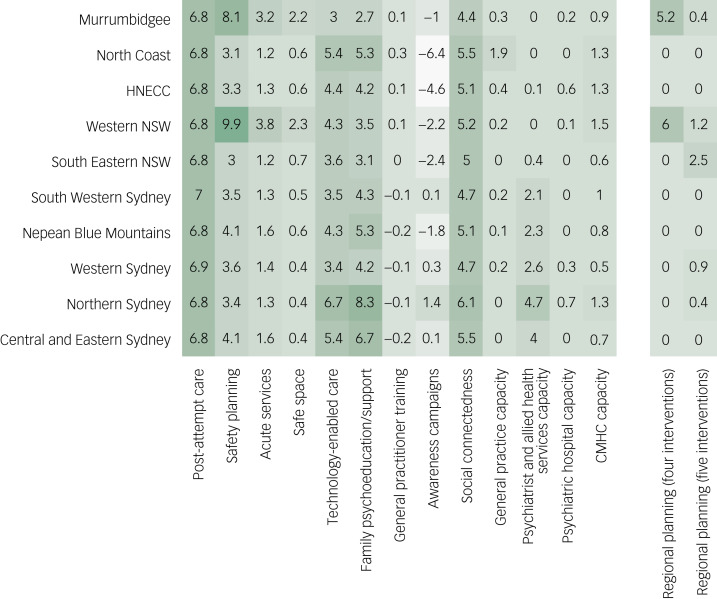
Percentage reductions in cumulative numbers of suicides over the period 2021–2031 projected under scenarios in which the 13 interventions in Supplementary Table 2 are implemented separately in each Primary Health Network (PHN) catchment (left panel). The panel on the right shows percentage reductions in projected numbers of suicides observed when the optimal combinations of four and five interventions for each PHN catchment are compared with the optimal state-level intervention combinations (combinations of interventions minimising state-level suicide mortality when implemented in all PHN catchments). Where the optimal combination of interventions at the PHN level is the same as the optimal state-level intervention combination, the percentage reduction in suicide mortality due to regional planning is (necessarily) equal to zero. CMHC, community mental healthcare services; HNECC, Hunter New England and Central Coast; NSW, New South Wales.

Figure 1 shows reductions in projected numbers of suicides for scenarios in which the modelled interventions are implemented separately in each PHN catchment. Post-suicide attempt care and social-connectedness programmes both yield more or less uniform reductions in suicide mortality across PHN catchments, preventing, respectively, 6.6–7% and 4.4–6.1% of suicides projected under the baseline (i.e. business as usual) scenario. Percentage reductions in projected numbers of suicides for technology-enabled care coordination (3.0–6.7%) and family psychoeducation and support programmes (2.7–8.3%) vary considerably among PHN catchments, while safety planning yields substantially greater percentage reductions in projected suicide mortality in the Western NSW PHN catchment (9.9%) and Murrumbidgee PHN catchment (8.1%) than in other PHN catchments (3.0–4.1%). Psychiatrist and allied health services capacity increases have limited (if any) impact on projected numbers of suicides in the South Eastern NSW PHN, Western NSW PHN, Hunter New England and Central Coast PHN, North Coast PHN and Murrumbidgee PHN catchments (maximum percentage reduction 0.4%), but prevent 2.1–4.7% of projected suicides in the remaining PHN catchments. Awareness campaigns similarly have widely varying impacts on projected suicide mortality across PHN catchments, significantly increasing numbers of suicides (by up to 6.4%) in several catchments, while preventing up to 1.4% of suicides in other catchments.

**Fig. 2:**
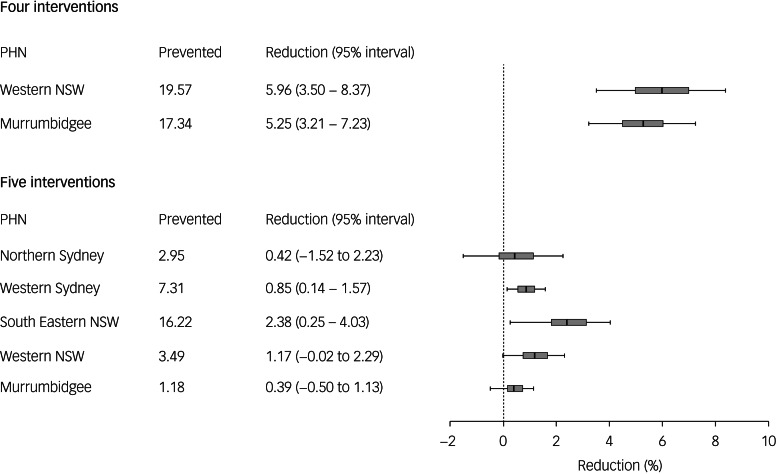
Projected reductions in total numbers of suicides over the period 2021–2031 observed when the optimal combinations of four and five interventions at the Primary Health Network (PHN) level are compared with the optimal state-level intervention combinations (see Table 1). Mean percentage reductions and 95% intervals reported in the rightmost column were derived from the distributions of projected numbers of suicides calculated in the sensitivity analyses. Note that the 95% intervals provide a measure of the impact of uncertainty in the assumed intervention effects, but should not be interpreted as confidence intervals. Mean percentage reductions and 50% and 95% intervals are plotted on the right. NSW, New South Wales.

The optimal state-level combination of four interventions includes post-attempt care, technology-enabled care coordination, family psychoeducation and support programmes, and social-connectedness programmes (Table 1); this intervention combination prevents 20.3% of 10 312 suicides projected under the baseline scenario over the 10-year forecast period (i.e. 1 January 2021 to the start of 2031) and is the best-performing combination of interventions for all PHNs except the Western NSW PHN and Murrumbidgee PHN. A combination of post-attempt care, safety planning, community-based acute care services and social-connectedness programmes yields the greatest reductions in suicide mortality in the Western NSW PHN and Murrumbidgee PHN catchments, preventing, respectively, 6% (95% interval 3.5–4.1%) and 5.2% (95% interval 3.2–7.2%) of suicides projected under the optimal state-level intervention scenario (Table 1, Fig. 2). The most effective state-level combination of five interventions, including post-attempt care, safety planning, technology-enabled care coordination, family psychoeducation and support programmes, and social-connectedness programmes, prevents 22.9% of suicides projected under the business as usual scenario. A combination of post-attempt care, technology-enabled care coordination, family psychoeducation and support programmes, awareness campaigns and social-connectedness programmes performs better than the optimal state-level combination in the South Eastern NSW PHN catchment, preventing an additional 16.2 suicides (95% interval 1.7–27.3) over the forecast period (Table 1 and Fig. 2).

**Table 1:** Best-performing combinations of interventions at the state and PHN levels (i.e. intervention combinations minimising the number of suicides projected over the period 2021–2031)

State-level interventions	PHN-level interventions		PHN(s)
Four interventions			
Post-attempt care, technology-enabled care, family psychoeducation, social connectedness	1	Post-attempt care, safety planning, acute care services, social connectedness	Western NSW, Murrumbidgee
Five interventions			
Post-attempt care, safety planning, technology-enabled care, family psychoeducation, social connectedness	1	Post-attempt care, technology-enabled care, family psychoeducation, awareness campaigns, social connectedness	Northern Sydney, South Eastern NSW
	2	Post-attempt care, technology-enabled care, family psychoeducation, social connectedness, psychiatrist and allied health services capacity increase	Western Sydney
	3	Post-attempt care, safety planning, family psychoeducation, awareness campaigns, social connectedness	Western NSW
	4	Post-attempt care, safety planning, acute care services, technology-enabled care, social connectedness	Murrumbidgee

Optimal PHN-level intervention combinations are presented only where they differ from the optimal state-level combinations. PHN, Primary Health Network; NSW, New South Wales.

**Fig. 3:**
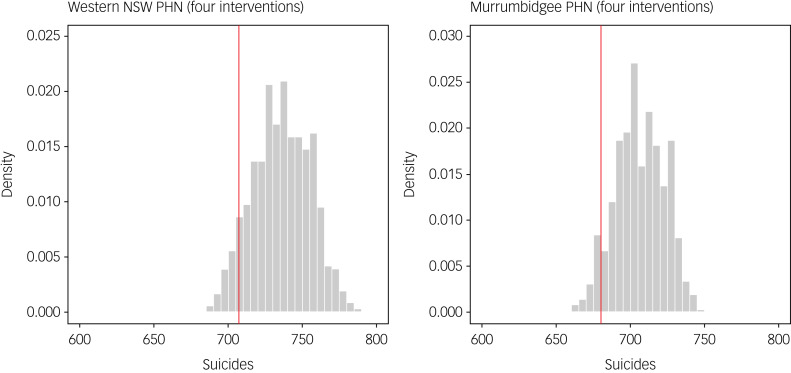
Distributions of total (cumulative) numbers of suicides projected for the Western New South Wales (NSW) and Murrumbidgee Primary Health Network (PHN) catchments over the period 2011–2031 under all (715) possible combinations of four interventions selected from the 13 interventions in Supplementary Table 2. The red vertical lines correspond to total numbers of suicides projected under the optimal state-level combination of interventions (i.e. the combination of interventions minimising state-level suicide mortality when implemented in all PHN catchments).

More than 90% of all possible combinations of four interventions selected from the 13 interventions modelled prevent fewer suicides than the optimal state-level intervention combination in the Western NSW PHN catchment (666 out of 715 possible intervention combinations; 93.1%) and Murrumbidgee PHN catchment (665 out of 715 combinations; 93%) (Fig. 3). Only one of the 1287 possible combinations of five interventions (i.e. 0.08%) is more effective than the optimal state-level combination in the South Eastern NSW PHN catchment.

## Discussion

The simulation results presented here indicate that while some suicide prevention and mental health services interventions have consistent, relatively large effects on suicidal behaviour (e.g. post-suicide attempt care, social-connectedness programmes), the efficacy of other interventions varies considerably across PHN catchments. Providing safety plans and follow-up care to suicidal patients who present to emergency departments, for example, is substantially more effective in the Western NSW PHN and Murrumbidgee PHN catchments than in other PHN catchments, due primarily to regional differences in per capita emergency department presentation rates (per capita presentation rates are higher in the Western NSW PHN and Murrumbidgee PHN catchments, effectively increasing the reach of safety planning in these catchments). The potential dependence of intervention effects on local context has significant implications for the development of multi-component suicide prevention programmes, as combinations of interventions that are optimal at a state or national level will not necessarily be optimal at a regional level. Our analyses suggest that the impact of independent regional planning (i.e. compared with centralised planning) on suicidal behaviour may be substantial, in some cases approaching that of implementing intensive post-attempt care (see Fig. 1). However, the capacity of suboptimal commissioning decisions to severely limit any potential benefit of local programme development is high; many possible combinations of the interventions modelled here prevent substantially fewer suicides than the optimal state-level combinations in those PHN catchments where optimal regional planning has a significant impact on projected suicide mortality (see Fig. 3).

The potentially substantial regional differences in the effects of individual suicide prevention measures observed in our simulation analyses (see Fig. 1) are consistent with the results of a previous analysis examining the impacts of selected mental health services interventions on suicide mortality in England and Wales.^11^ While et al. reported significantly greater decreases in suicide rates associated with the implementation of multiple suicide prevention policies in larger mental health services (those reporting higher numbers of patient contacts per year) and services providing care to socioeconomically deprived populations, although the effects of implementing alternative sets of polices in these (and other) services were not considered.^11^ As far as we are aware, the analyses presented here are the first to quantify the potential impact of independent regional planning (relative to national- or state-level planning) on the effectiveness of suicide prevention programmes. Our modelling provides conditional support for the Australian Government's decision, made in response to the National Mental Health Commission's 2014 National Review of Mental Health Programmes and Services, to devolve responsibility for the commissioning of suicide prevention services to the (then) recently established PHNs.^19^ To the extent that it enables optimal tailoring of multi-component suicide prevention programmes to the specific needs of local communities and services, this devolution of responsibility for suicide prevention planning has the potential to significantly reduce suicidal behaviour at a regional level (see Figs 1 and 2).

Although the transition to regional commissioning of mental health and suicide prevention services in Australia potentially facilitates the development of locally optimal suicide prevention programmes, the results of our analyses indicate that this will require that PHNs are provided with the necessary resources and tools to support reliable, evidence-based decision-making. The National Mental Health Service Planning Framework (NMHSPF) and NMHSPF Planning Support Tool, which originated with Australia's Fourth National Mental Health Plan (2009–2014), were developed to guide regional mental health services planning decisions, providing estimates of future demand for mental healthcare and the resources (mix of service types, workforce, funding, etc.) needed to meet that demand.^20^ More recently, we have applied participatory modelling methods to develop interactive dynamic simulation models designed specifically to support regional commissioning and coordination of suicide prevention services.^16,21,22^ These models provide a logically consistent ‘what-if’ tool that can be used to improve understanding of the possible impacts of proposed initiatives before they are implemented in the real world, and, unlike the NMHSPF and other static models, are capable of capturing highly complex system behaviour resulting from feedback, delayed effects and intervention interactions.^23,24^ Routine use of advanced decision support tools such as these in suicide prevention planning is likely to be critical if the potentially significant reductions in suicide mortality attainable through local programme development are to be realised.

### Limitations

This study has a number of important limitations that should be noted. First, the results presented here depend on the specific suicide prevention and mental health services interventions included in the simulations and the particular distribution of demographic, health and services system characteristics in our study population, so the external validity of our conclusions is potentially limited. Analyses examining the effects of independent regional suicide prevention planning in other contexts (i.e. different interventions and populations) are needed to determine the range of conditions under which local programme development may be expected to contribute to significant decreases in suicide mortality. Second, we have only considered the potential impacts of implementing optimal combinations of suicide prevention and mental health services interventions in each PHN catchment; however, independent local planning may increase the effectiveness of suicide prevention programmes in other ways, including through greater stakeholder engagement and by providing increased opportunity for identifying promising new initiatives and improved approaches to implementation.^25,26^ Potential reductions in suicidal behaviour resulting from a shift to autonomous regional planning may therefore be significantly greater than our modelling suggests. Finally, the simulation analyses presented here effectively disregard the substantial adverse mental health impacts of social dislocation and unemployment resulting from the ongoing COVID-19 pandemic.^27,28^ Nevertheless, preliminary analyses indicate that incorporating the effects of COVID-19 on psychological distress and suicidal behaviour in our simulations would not significantly affect our principal conclusions (see Supplementary Material).

In conclusion, the simulation modelling analyses presented here indicate that the effectiveness of individual suicide prevention measures may depend substantially on the specific populations and services systems in which those measures are implemented, so that evidence-based, multi-component suicide prevention programmes developed at a national or state level will not necessarily be optimal at a regional level. Potentially substantial reductions in local suicide mortality, comparable to those projected for effective clinical interventions (e.g. post-attempt care; see Fig. 1), may be achievable by devolving responsibility for the commissioning of mental health and suicide prevention services implemented as part of a national suicide prevention strategy to regional health authorities. Nevertheless, our results also indicate that the capacity of suboptimal commissioning decisions to limit any potential benefit of local programme development is high, emphasising the need to ensure that regional planners are provided with the resources and tools required to support reliable, evidence-based decision-making. Dynamic simulation models, co-developed with local stakeholders using participatory modelling methods, offer perhaps the most promising means of addressing the challenge of developing regional suicide prevention programmes that are consistent with the best available scientific evidence while also accommodating heterogeneity in local services and community needs.

## Data Availability

The authors confirm that the data supporting the findings of this study are available within the article and its supplementary materials. Details of all data sources used for model parameterisation and calibration are provided in the Supplementary Material.

## Supplementary material

For supplementary material accompanying this paper visit http://dx.doi.org/10.1192/bjo.2021.989. click here to view supplementary material
