# Supplementary material for: Regional suicide prevention planning: a dynamic simulation modelling analysis
Source: BJPsych Open. 2021 Aug 31;7(5):e157. doi: 10.1192/bjo.2021.989 (PMC8444054; doi:10.1192/bjo.2021.989)
Supplement: Supplementary file 1 [file S2056472421009893sup001.docx]

Supplementary material. Regional suicide prevention planning: a dynamic simulation modelling analysis.

1. Primary Health Networks included in the analyses

Table S1. Descriptive statistics for the Primary Health Network (PHN) catchments included in the simulation modelling analyses.

| Primary Health Network (PHN) | Population | Area (km^2^) | Population density (per km^2^) | Disadvantaged (%) | Suicide rate (per 10^5^) |
| --- | --- | --- | --- | --- | --- |
|  |  |  |  |  |  |
| Central and Eastern Sydney | 1572237 | 626 | 2511.56 | 5.61 | 9.10 |
| Northern Sydney | 914229 | 890 | 1027.22 | 0 | 7.92 |
| Western Sydney | 948593 | 766 | 1238.37 | 22.22 | 7.61 |
| Nepean Blue Mountains | 367783 | 9063 | 40.58 | 15.00 | 10.98 |
| South Western Sydney | 964351 | 6186 | 155.89 | 44.17 | 7.99 |
| South Eastern NSW | 611202 | 50177 | 12.18 | 16.44 | 11.71 |
| Western NSW | 307402 | 433379 | 0.71 | 34.59 | 10.39 |
| Hunter New England and Central Coast | 1247225 | 130646 | 9.55 | 22.13 | 11.37 |
| North Coast | 513379 | 32047 | 16.02 | 27.25 | 12.18 |
| Murrumbidgee | 242867 | 124413 | 1.95 | 9.11 | 11.58 |
|  |  |  |  |  |  |

2. Model structure

*2.1. Overview*

The core model structure, replicated for each of the 10 New South Wales (NSW) PHNs, incorporates a set of interconnected sub-models or sectors, including: 1) a population sector, capturing changes in catchment population size resulting from births, migration, and mortality; 2) a psychological distress sector that models flows of people to and from states of low psychological distress (i.e., Kessler 10 [K10] score 10−15) and moderate to very high psychological distress (K10 score 16−50); 3) a mental health services sector, modelling the movement of distressed patients through a network of possible service pathways involving general practitioners, psychiatrists and allied health care providers, psychiatric inpatient care, community mental health care services, and online services; and 4) a suicidal behaviour sector that captures numbers of self-harm hospitalisations and suicides. Figure S1 presents a high-level map of the core model showing the connections among sectors.


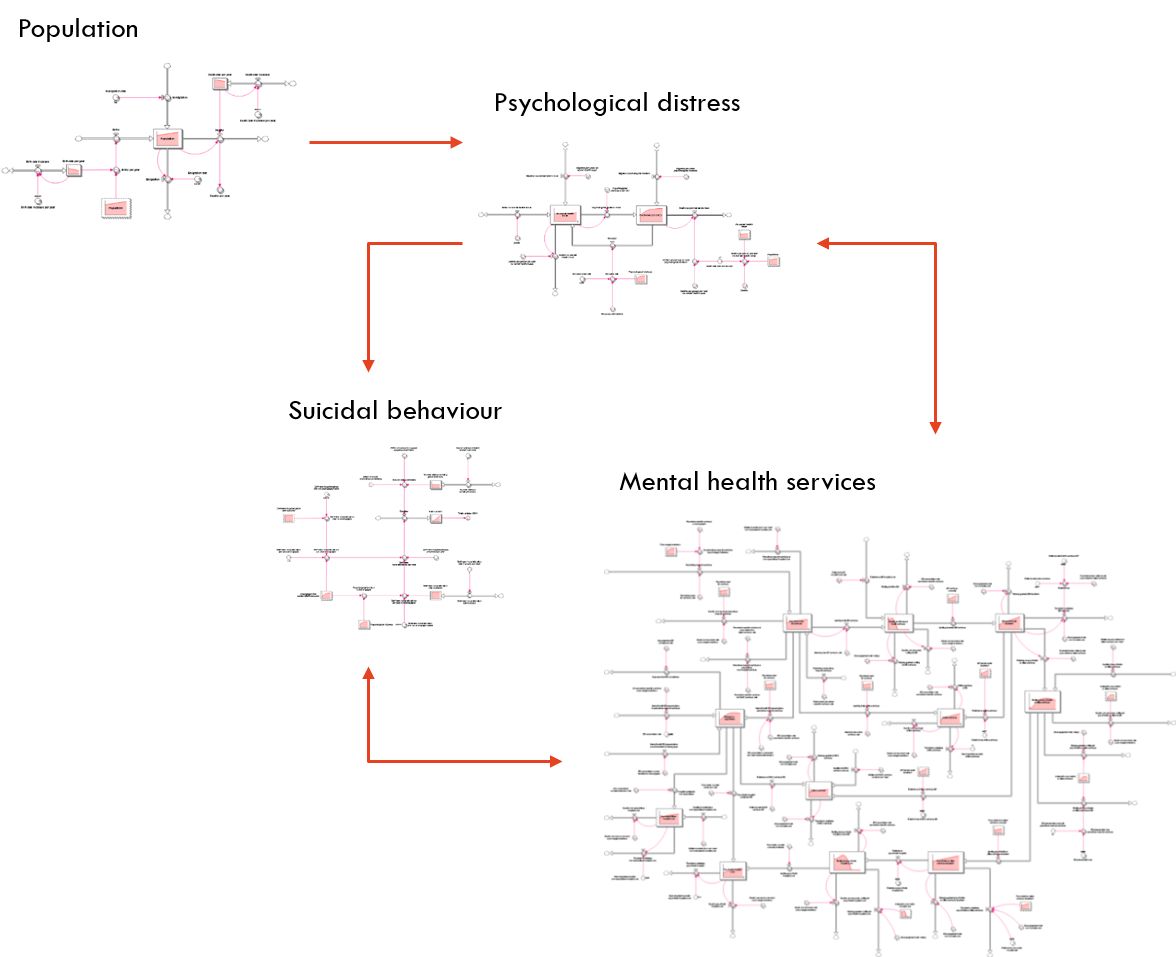


Figure S1. High-level map of the core system dynamics model showing the causal connections among model sectors. Single-headed arrows indicate unidirectional causal connections; bidirectional causal connections are shown as double-headed arrows.

*2.2. Population sector*

Figure S2 shows the structure of the population sector, which captures changes in catchment population size due to births, migration, and mortality. The total population of each PHN catchment is represented as a single stock that increases through births and immigration and decreases via mortality and emigration. Births and deaths occur at yearly rates $bP$ and $mP$, respectively, where $P$ is the population size and the per capita birth rate $b$ and per capita mortality rate $m$ increase or decrease by constant fractions each year. Net migration is equal to $I-eP$, where $I$ is total immigration per year and $e$ is the per capita emigration rate per year. Population estimates derived from the system dynamics model and from HealthStats NSW (http://www.healthstats.nsw.gov.au/) are plotted in figure S3.


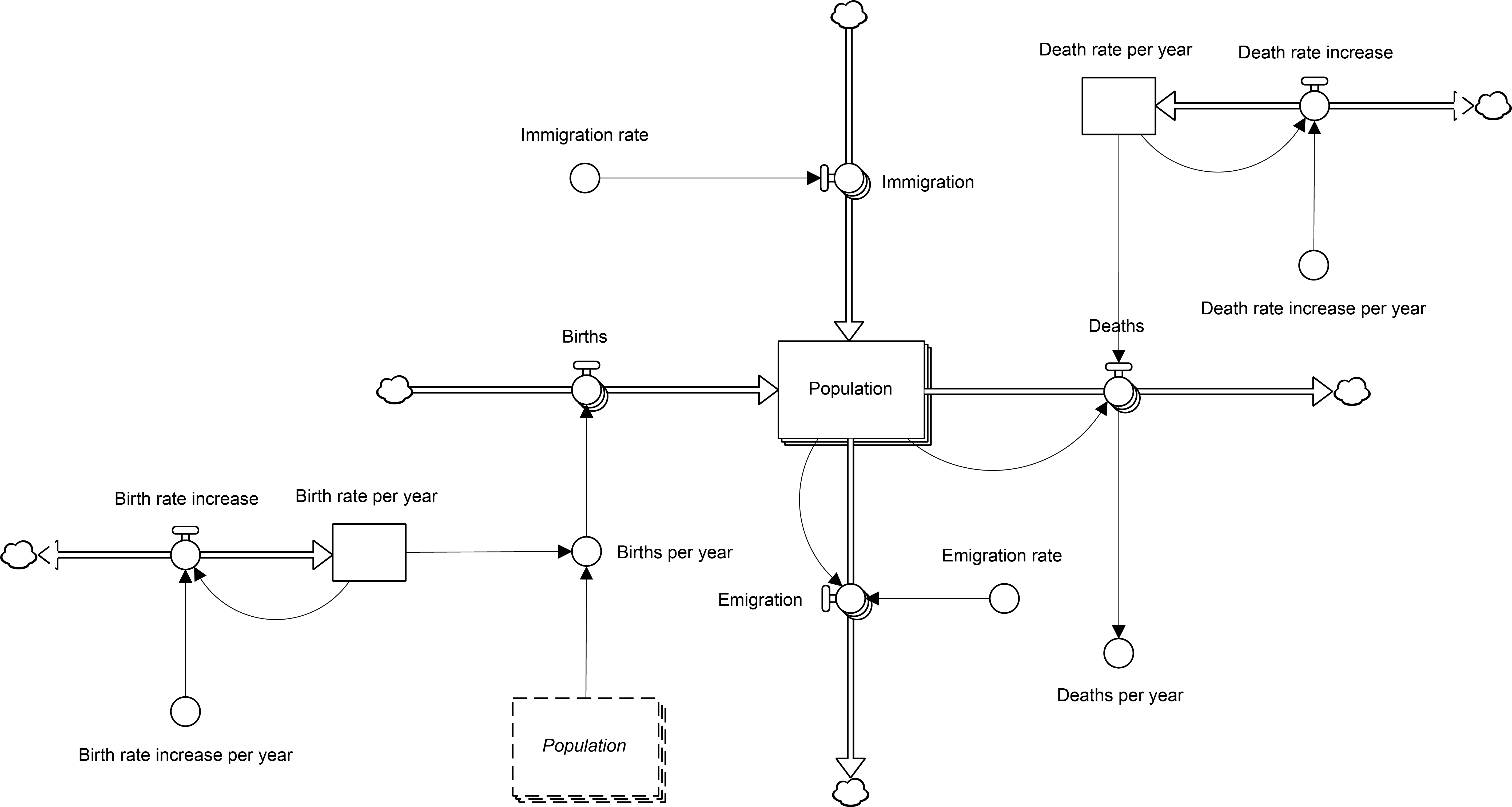


Figure S2. Structure of the population sector.

*2.3. Psychological distress sector*

The psychological distress sector captures transitions between states of low psychological distress (K10 scores 10−15) and moderate to very high psychological distress (K10 scores 16 and above) in each PHN catchment. People with no mental health issue (i.e., low psychological distress) and those experiencing moderate to very high levels of psychological distress are modelled as stocks connected by flows corresponding to psychological distress incidence and recovery (see figure S4). Psychological distress incidence is equal to $sL$, where $s$ is the


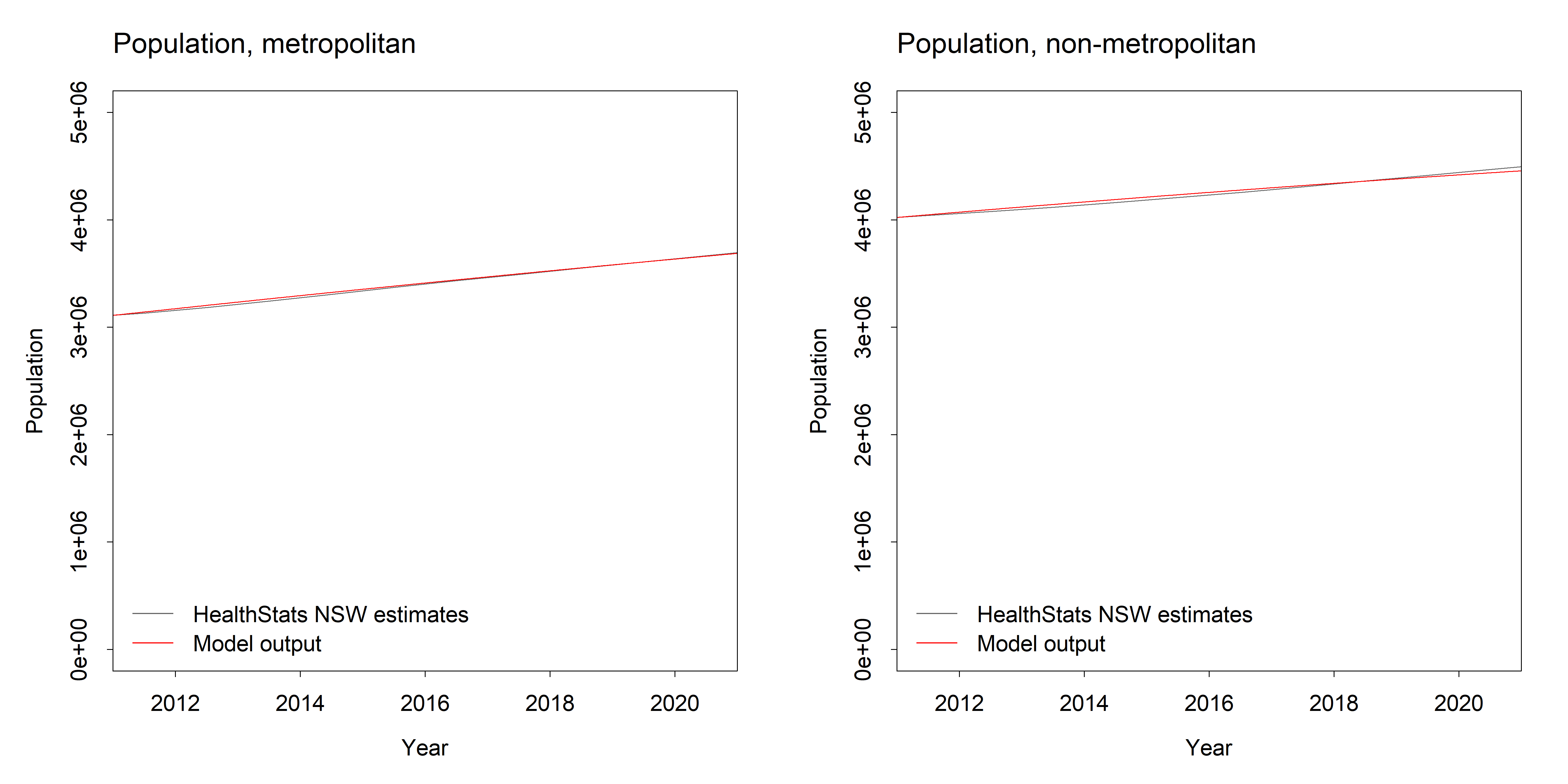


Figure S3. Population estimates (metropolitan and non-metropolitan) derived from the system dynamics model and from HealthStats NSW (http://www.healthstats.nsw.gov.au/). Metropolitan estimates are for Central and Eastern Sydney PHN, Northern Sydney PHN, and Western Sydney PHN; non-metropolitan estimates are for all remaining PHNs.

per capita rate of psychological distress onset per year and $L$ is the number of people in the population experiencing low psychological distress. People with moderate to very high psychological distress recover at a rate equal to $rH+T$, where $r$ is the per capita spontaneous recovery rate per year, $H$ is the number of moderately to highly distressed people in the population, and $T$ is the number of people moving from a state of higher psychological distress to a state of low psychological distress per year due to effective mental health treatment (see section 1.4 below).

Numbers of people in each psychological distress state increase or decrease through net migration at rates $p_{i}I-q_{i}eP$, where $p_{i}$ and $q_{i}$ are the proportions of people with psychological distress level $i$ (low or moderate to very high) in the total NSW population and the PHN catchment population, respectively, $I$ is the total number of people immigrating into the PHN catchment per year, $e$ is the per capita emigration rate per year, and $P$ is the size of the PHN catchment population. As high-prevalence mental disorders generally originate during adolescence and early adulthood (e.g., Weissman et al., 1996; Michael et al., 2007), children are assumed to be born without a mental health issue, so that births are added to the stock of people with a low level of psychological distress (at a rate $bP$ per year; see section 1.2). Per capita mortality among people with moderate to very high psychological distress is assumed to be 1.27 times that among people experiencing low psychological distress based on Russ et al. (2012). Figure S5 presents estimates of the prevalence of moderate to very high psychological distress derived from the simulation model and the NSW Population Health Survey (see http://www.healthstats.nsw.gov.au/).


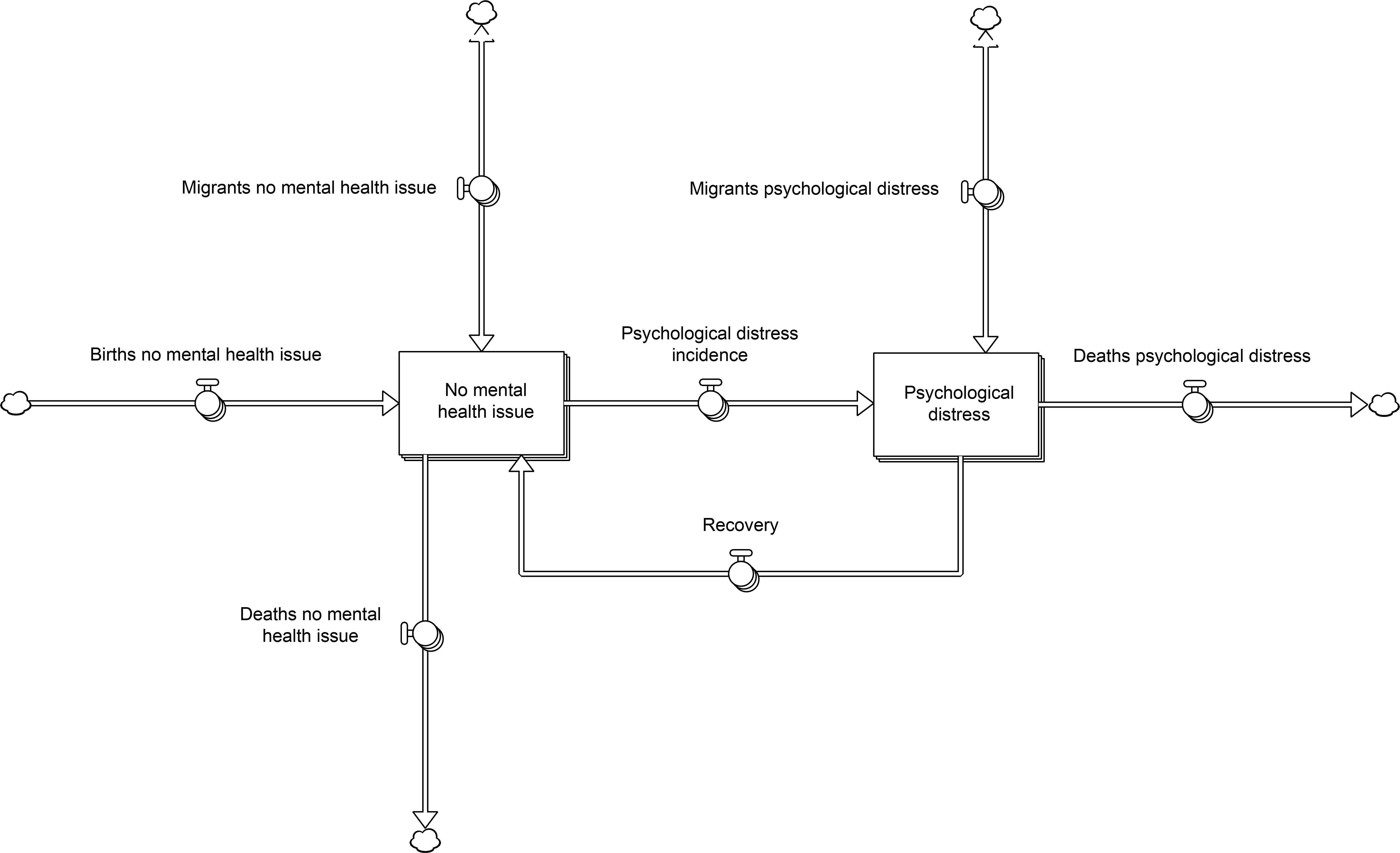


Figure S4. Stock and flow structure of the psychological distress sector.

*2.4. Mental health services sector*

Figure S6 presents a high-level map of the mental health services sector, which models flows of psychologically distressed patients through the mental health care system. People with moderate to very high levels of distress engage with mental health services in one of two ways; they may perceive a need for mental health care and seek help (e.g., from a general practitioner or online services), or they may present to an emergency department (e.g., for self-harm) without having previously perceived a need for treatment. After engaging with mental health services, patients may recover following treatment, returning to the stock of people with no mental health issue, be treated but not recover, or disengage due to excessive waiting times (a consequence of insufficient services capacity) or because they are dissatisfied with the care they receive. Patients who are treated but do not recover return to perceiving a need for services and will eventually seek help again if they do not recover


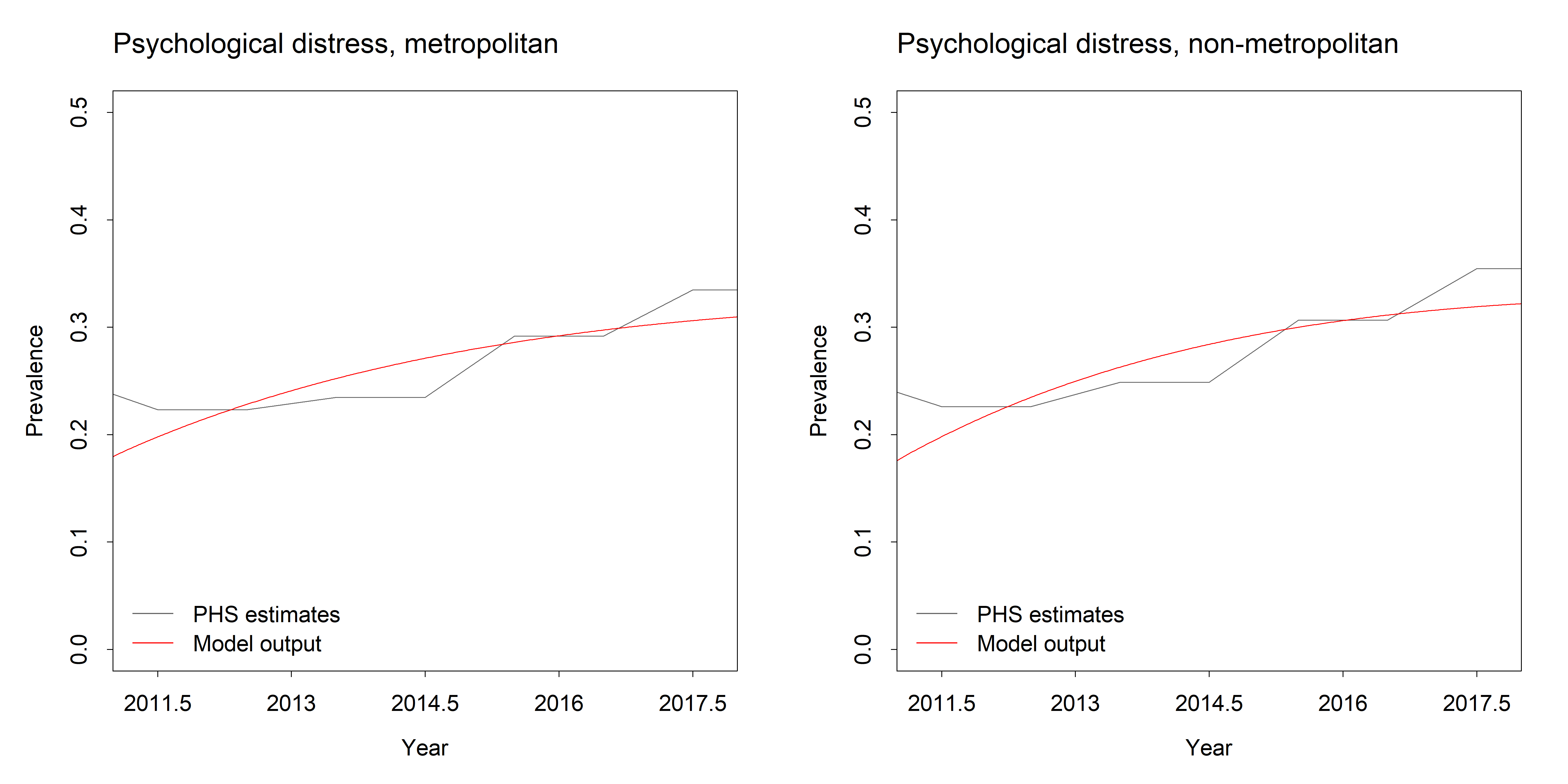


Figure S5. Psychological distress prevalence estimates (metropolitan and non-metropolitan) derived from the system dynamics model and corresponding NSW Population Health Survey (PHS) estimates. Metropolitan estimates are for Central and Eastern Sydney PHN, Northern Sydney PHN, and Western Sydney PHN; non-metropolitan estimates are for all remaining PHNs.

spontaneously; psychologically distressed people entering the mental health services system therefore continue receiving treatment (modelled as individual service contacts; see below) until they recover, disengage, or die (mortality is captured in the model, but is not shown in figure S6 for simplicity). The principal components of the mental health services sector are described in detail below.

*Help seeking* — People experiencing moderate to very high levels of distress who are not considering engaging with mental health services perceive a need for care at a rate equal to $pD$, where $D$ is the number of moderately to highly distressed people not currently considering treatment and $p$ is the per capita rate that people with moderate to very high psychological distress perceive a need for care per year. The per capita rate $p$ is assumed to increase at a constant rate per year due to increasing public awareness of high-prevalence mental disorders and available treatment options. After perceiving a need for treatment, people engage with mental health services at a per capita rate that also increases linearly over time (see *GP and online services* below). Recently treated patients who have not recovered or disengaged from services return to perceiving a need for care and may attend subsequent (planned or unplanned) appointments with a GP or community-based psychiatric services, be admitted to a general hospital, commence online treatment, or present to an emergency department;


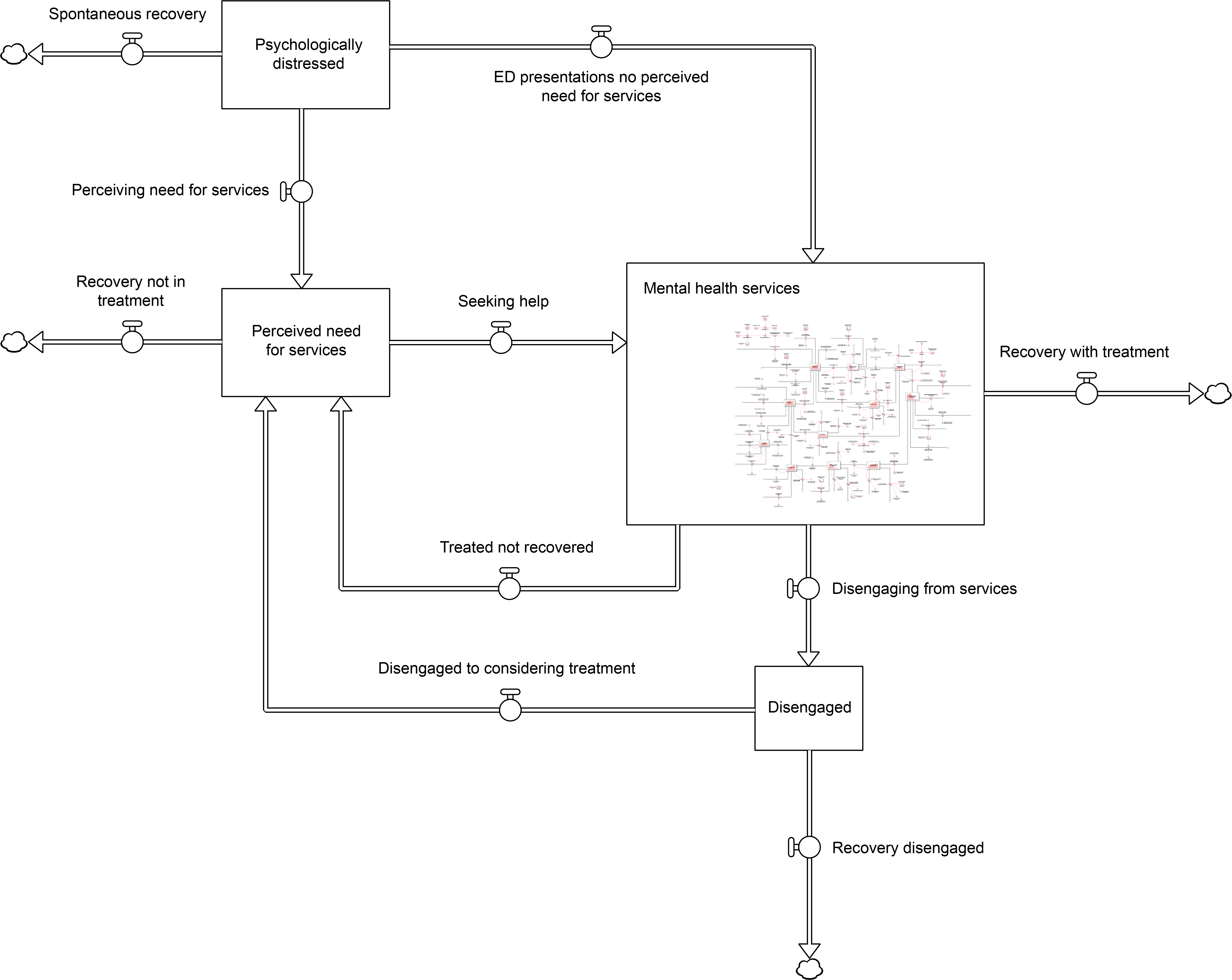


Figure S6. High-level map of the mental health services sector.

the stock of people perceiving a need for services (see figure S7) therefore contains a mix of prospective patients and patients already engaged with the mental health care system. These prospective and current patients are assumed to experience the same per capita mortality and (spontaneous) recovery rates as moderately to highly distressed people who are not considering treatment.

*GP and online services* — People with a perceived need for mental health services seek help from a GP at a rate $sP$, where $P$ is the number of psychologically distressed people perceiving a need for care (those in the stock labelled ‘Perceived need for services’ in figure S7) and $s$ is the per capita rate at which people with a perceived need for care seek help from a GP per year. The per capita rate $s$ is assumed to increase at a constant rate per year, increasing the help seeking rate among people who perceive a need for treatment. Prior to receiving care, patients seeking help from a GP or referred to GP services after completing hospital inpatient care wait for a


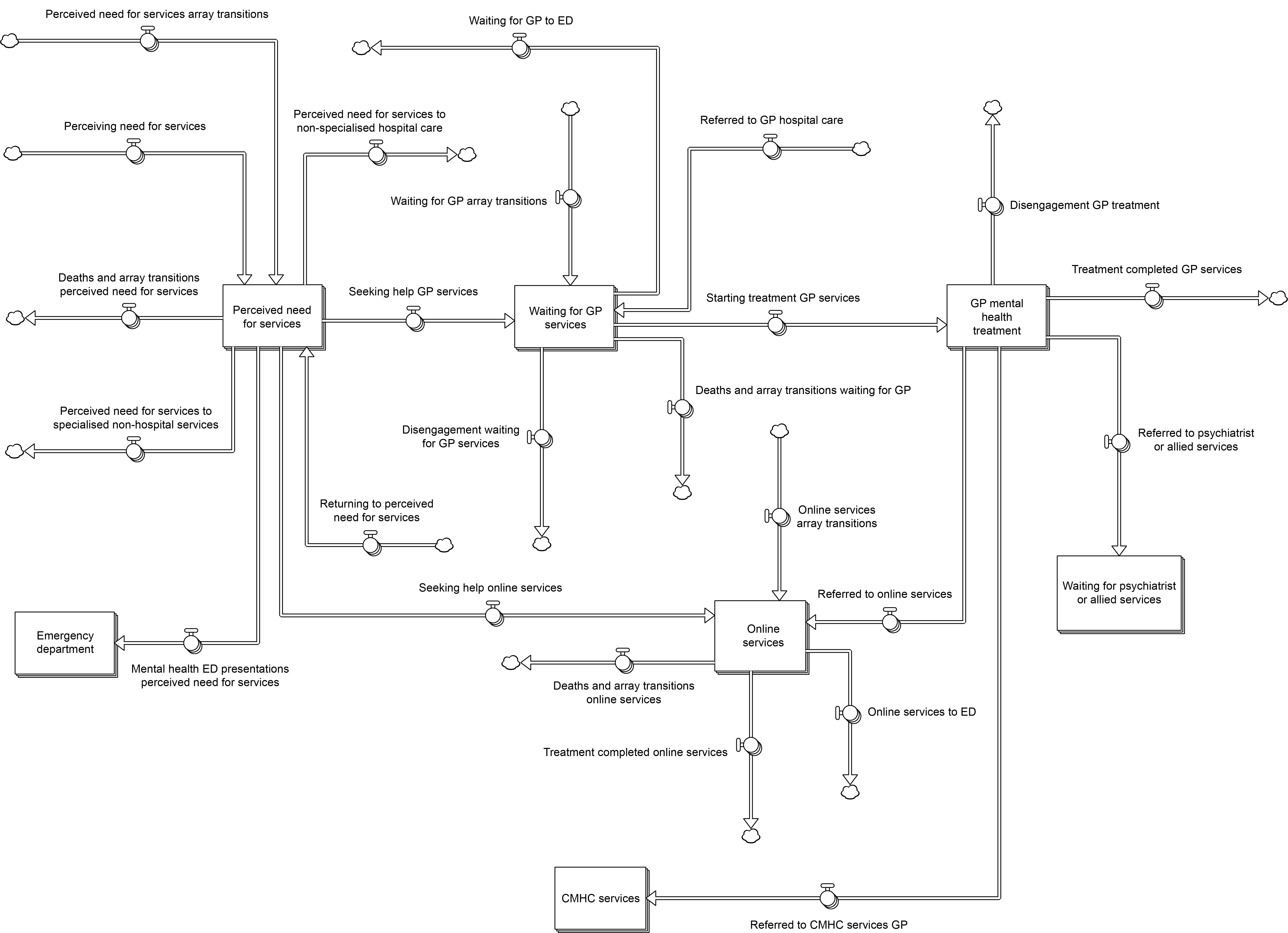


Figure S7. Stock and flow structure of the help-seeking, general practitioner (GP) services, and online services components of the mental health services sector.

varying period of time that depends on services capacity and the total number of patients waiting for care. GP services capacity, i.e., the number of mental health-related GP consultations that can be provided per year, is assumed to increase at a constant rate per year, estimated from Medicare Benefits Schedule (MBS) claims data for the period 2012−2017 (see figure S8). Mental health-related GP consultations are represented as a stock with outflows corresponding to treatment completion, referral to other services (including psychiatrist and allied health services, state-funded community mental health care services, and online services), and disengagement resulting from dissatisfaction with the care provided. The recovery rate among patients completing treatment is equal to $vrT$, where $T$ is the number of patients completing treatment per year, $v$ is the proportion of patients completing treatment who receive psychological therapy, and $r$ is the proportion of patients who recover after receiving psychological therapy. Patients completing treatment who do not recover return to the stock of people who perceive a need for care.

Treatment commencement rates for online (self-help) services in each catchment are equal to $hP+uG$, where $P$ is the number of moderately to highly distressed people who perceive a need for care (i.e., those in the stock labelled ‘Perceived need for services’ in figure S7), $h$ is the per capita rate that people with a perceived need for care access online services per year, $G$ is the GP services provision rate (i.e., the total number of patients attending a GP consultation per year), and $u$ is the fraction of patients visiting a GP for a mental health issue referred to online services. Prior to completing treatment, people accessing online services may recover spontaneously, present to an emergency department, or die (figure S7); treatment completion rates are equal to $O/d$, where $O$ is the number of people engaged in online treatment and $d$ is the mean duration of online treatment programs (6 weeks, or 0.115 years; see Christensen et al., 2004). People completing online treatment programs recover at a rate equal to ${zO}/d$, where $z$ is the proportion of psychologically distressed people completing treatment who return to a state of low psychological distress (assumed to be 0.4; see Christensen et al., 2004).

*Psychiatrist and allied health services* — The structure of the psychiatrist and allied health services component of the mental health services sector is shown in figure S9. Prior to receiving treatment, patients referred to psychiatrist or allied health services by a GP or after completing hospital inpatient care wait for a period of time that depends on services capacity and the number of patients waiting for care. The stock of people waiting for treatment also contains patients currently engaged with specialised services who have planned (follow-up)


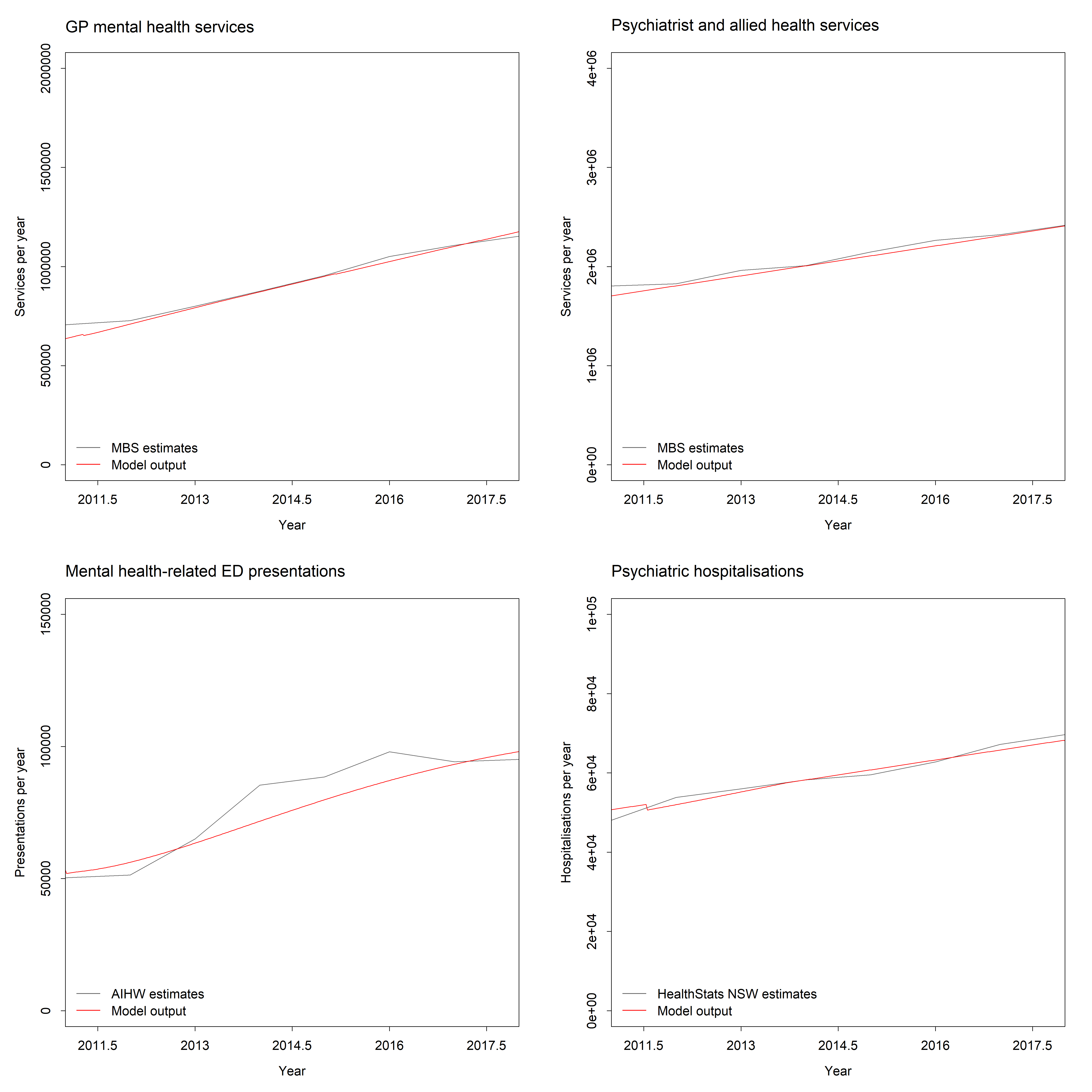


Figure S8. Mental health services usage rates (for all PHN catchments combined) derived from the system dynamics model and from Medicare Benefits Schedule (MBS) data, data published by the Australian Institute of Health and Welfare (AIHW), and data available from HealthStats NSW (http://www.healthstats.nsw.gov.au/).

appointments (these patients and patients referred to services after receiving hospital inpatient care enter via the flow labelled ‘Additional psychiatrist or allied health services’; see figure S9). Services capacity, i.e., the total number of psychiatrist and allied health services that can be provided per year, increases at a constant rate per year, estimated from MBS claims data for the period 2012−2017 (see figure S8). Patients receiving treatment are referred to psychiatric hospital services, disengage from the mental health services system due to dissatisfaction with the care provided, recover, or return to perceiving a need for care (these patients flow back into the stock labelled ‘Perceived need for services’; see figure S7). The recovery rate is equal to $rT$, where $T$ is the number of patients who complete treatment per year and $r$ is the fraction of patients recovering after receiving specialised psychiatric care. Psychiatrist and allied health services usage rates derived from the model and from MBS claims data are presented in figure S8.

*Hospital services* — Mental health-related hospital services captured in the model include psychiatric and non-specialised inpatient care, outpatient care delivered by community mental health care (CMHC) teams, and emergency department attendances (see figure S10). Emergency department (ED) presentations occur at a rate $eC+fN$, where $C$ is the number of people perceiving a need for care (including all patients waiting for care or engaged in online treatment and those who have disengaged from services; figures S7, S9−S11), $N$ is the number of psychologically distressed people who do not perceive a need for care, and $e$ and $f$ are per capita ED presentation rates for distressed people perceiving a need for care and with no perceived need for care, respectively. People presenting to an ED may be admitted to a psychiatric or general hospital ward, referred to CMHC services, or discharged to the community. A fraction of patients perceiving a need for care who are discharged to the community disengage from services due to dissatisfaction with the care provided. Figure S8 shows ED presentation rates derived from the model and from data published by the Australian Institute of Health and Welfare (2018).

Psychiatric and general (non-specialised) hospital admission rates are constrained by hospital capacity (i.e., the total numbers of patients that can be admitted annually), which is assumed to increase linearly over the simulation period; capacity increase rates per year were estimated from hospital separations data available from HealthStats NSW (see http://www.healthstats.nsw.gov.au/) and data published by the Australian Institute of Health and Welfare (2018). Psychiatrists refer highly distressed patients to psychiatric hospital care at a rate equal to $qM$, where $M$ is the number of patients receiving psychiatrist and allied health services per year and $q$ is the fraction of patients receiving psychiatrist and allied health services referred to psychiatric hospital care. Prior to being admitted, patients referred to a psychiatric hospital wait for a period of time that depends on hospital capacity, the number of patients waiting for care, and the rate at which patients are being admitted via EDs (available capacity declines as the ED-related admission rate increases). People with a perceived need for services are admitted for general hospital care without presenting to an ED at a per capita rate that remains constant over the simulation period (the flow labelled ‘Additional admissions non-specialised hospital care’ in


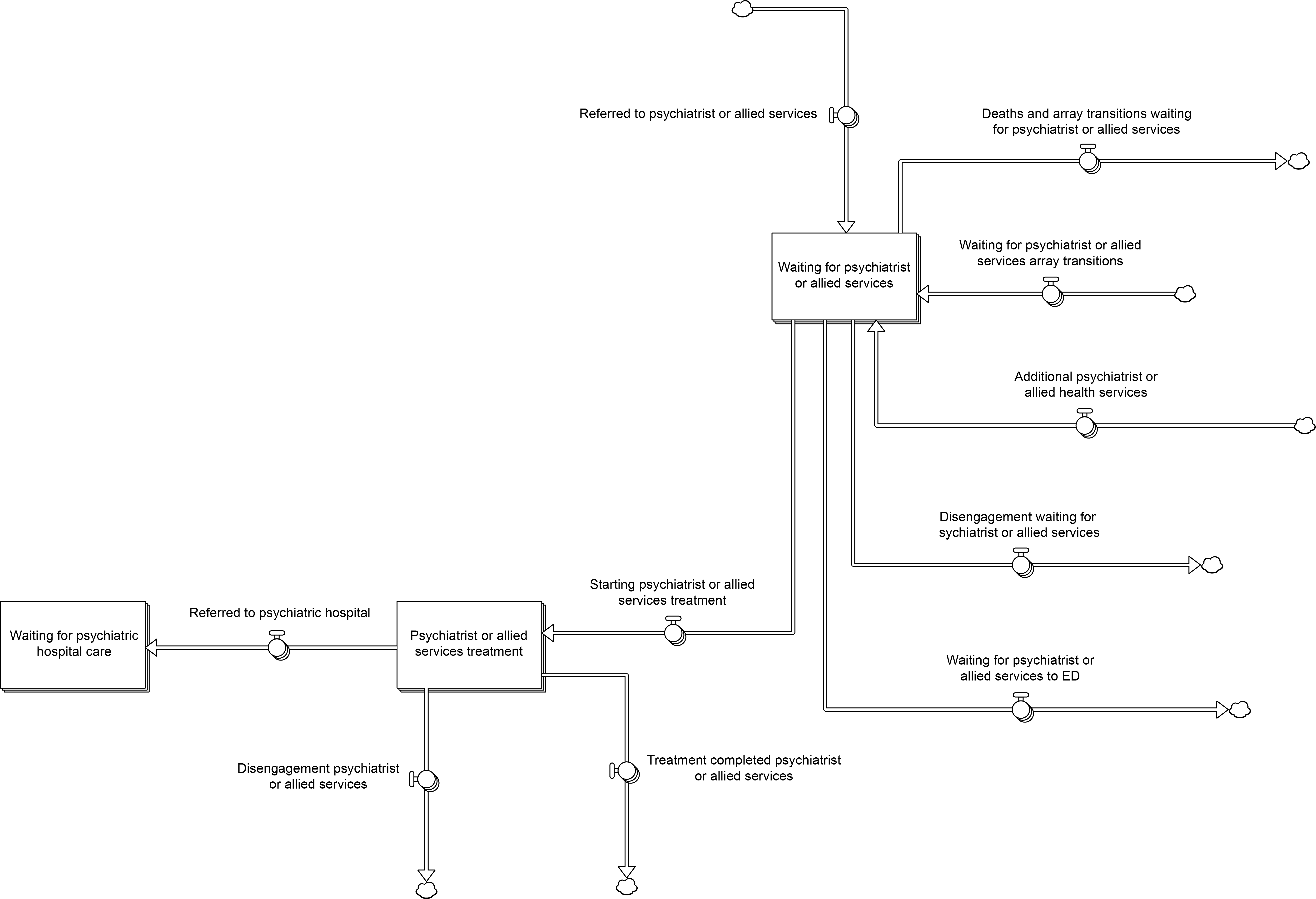


Figure S9. Stock and flow structure of the psychiatrist and allied health services component of the mental health services sector.


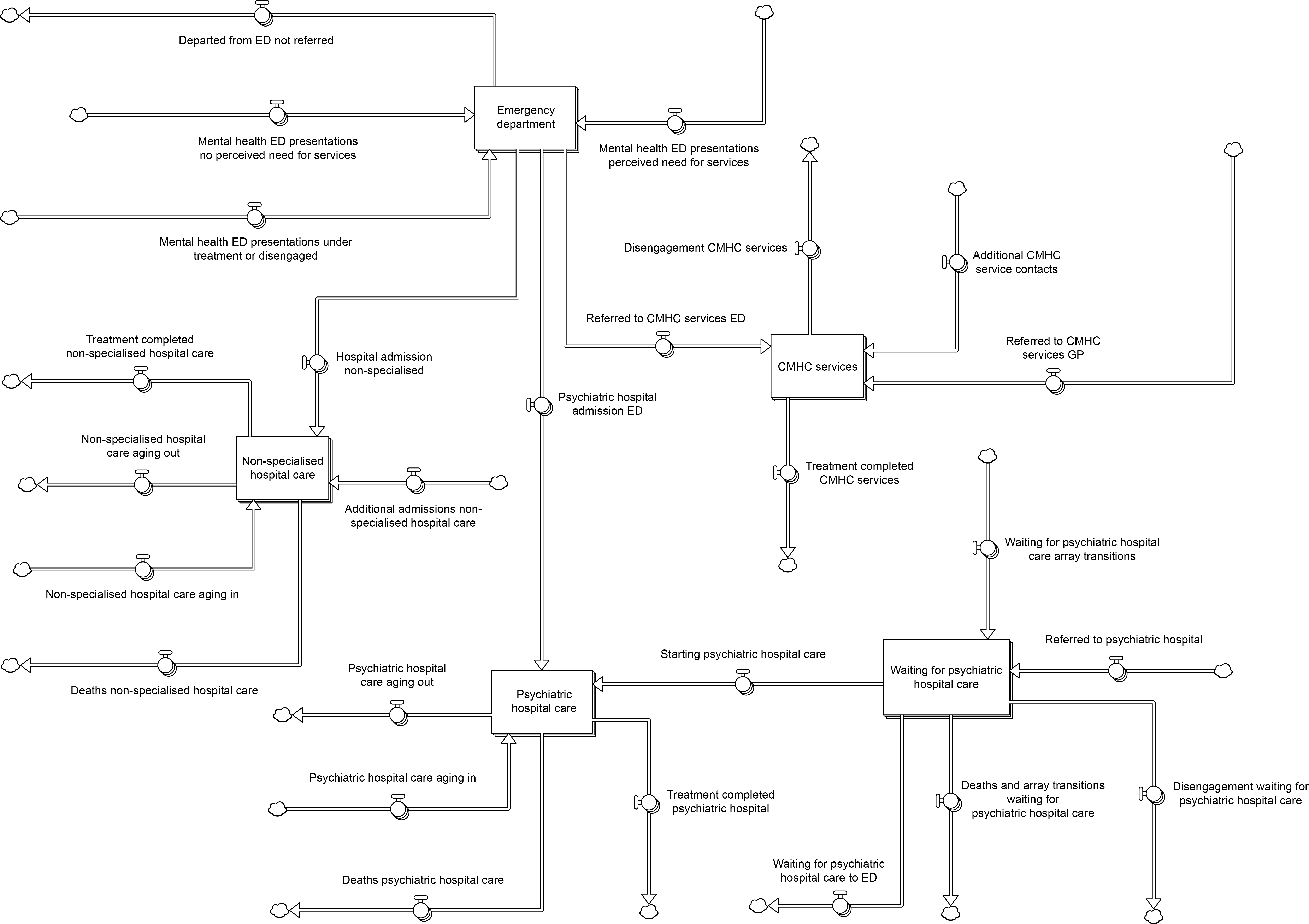


Figure S10. Stock and flow structure of the hospital services component of the mental health services sector.

figure S10). A fraction of patients discharged from psychiatric or general hospital care recover (psychiatric hospital care only) or disengage from services due to dissatisfaction with the care provided; patients who do not recover or disengage are referred to a GP, psychiatrist and allied health services, or CMHC services for follow-up care.

Community mental health care (CMHC, or hospital outpatient) services are provided at a rate $hG+pE+S+A+uP$, where $G$ is the number of patients visiting a GP per year, $h$ is the proportion of patients visiting a GP who are referred to CMHC services, $E$ is the number of people presenting to an ED per year who are not admitted to hospital, $p$ is the proportion of patients discharged from an ED referred to CMHC services, $S$ and $A$ are, respectively, the numbers of patients discharged from psychiatric and general hospital inpatient care per year referred to CMHC services, $P$ is the number of people with a perceived need for care (i.e., those in the stock labelled ‘Perceived need for services’; see figure S7), and $u$ is the per capita CMHC services contact rate among people with a perceived need for care (e.g., for follow-up appointments). Patients receiving CMHC services recover, return to the stock of people perceiving a need for services, or disengage due to dissatisfaction with the care provided (figure S10). The per-service recovery rate for patients who do not disengage from treatment is calculated as ${rK}/T$, where $K$ is the number of CMHC services that can be provided per year while maintaining the base (or reference) per-service recovery rate $r$ and $T$ is the current CMHC services provision rate; note that as the current services provision rate, $T$, increases relative to the reference capacity $K$, the per-service recovery rate, ${rK}/T$, declines (due to increased pressure on services). CMHC services capacity (i.e., $K$) is assumed to increase at a constant rate per year, estimated from services usage data published by the Australian Institute of Health and Welfare (2018).

*Disengagement* — Patients waiting for a GP, psychiatrist and allied health services, or psychiatric hospital care are assumed to disengage from the mental health care system at a constant per capita rate per year (estimated from data reported in Tyrer et al., 1995). The total disengagement rate therefore increases whenever the demand for mental health services exceeds services capacity, as the number of patients waiting for care continues to increase while patients are being referred to services at a higher rate than they can be treated. Patients receiving treatment also disengage from services due to dissatisfaction with the care provided (figures S7, S9, S10). Disengagement from the mental health care system is assumed to increase the risk of suicidal behaviour (due to a loss of hope that effective treatment is available, or trauma associated with inadequate care), so that an increase in the disengagement rate leads to an increase in self-harm hospitalisation and suicide death rates (see section 1.5 below). Patients who have disengaged from treatment return to the stock of people perceiving a need for care (i.e., they consider engaging with services again) at a constant per capita rate per year (figure S11).


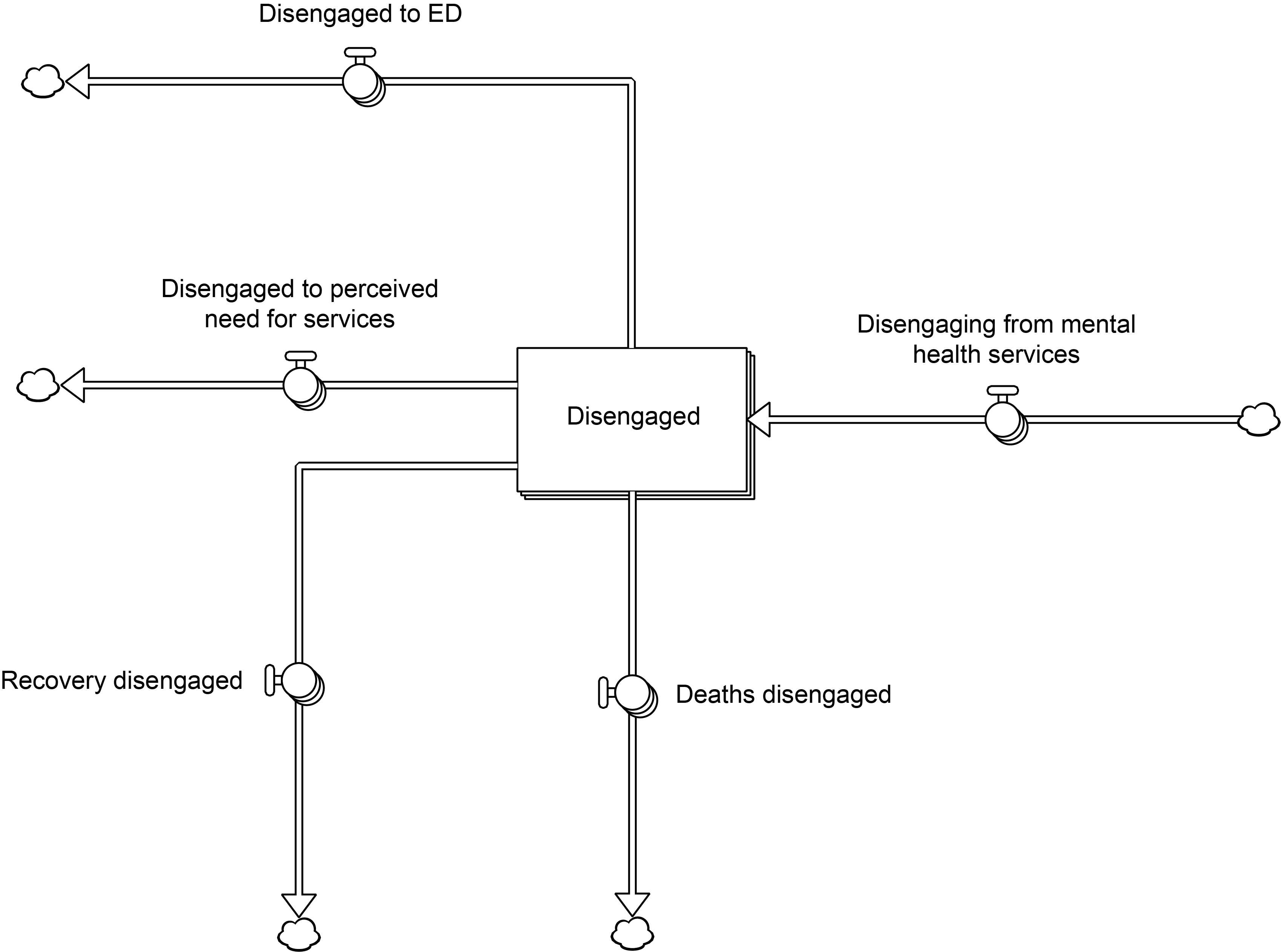


Figure S11. Stock and flow structure of the disengagement component of the mental health services sector.

*2.5. Suicidal behaviour sector*

Figure S12 presents the structure of the suicidal behaviour sector, which captures self-harm hospitalisations and suicide deaths (note that we equate suicide attempts with self-harm hospitalisations due to data availability constraints). The suicide attempt rate is equal to $s\left( D-R \right)+\eta sR$, where $D$ is the number of moderately to highly distressed people in the population, $R$ is the number of people disengaged from mental health services, $s$ is the per capita suicide attempt rate for psychologically distressed people who have not disengaged from services, and the suicide attempt rate ratio $\eta$ is assumed to be greater than 1 (i.e., the per capita attempt rate for people who have disengaged from services is assumed to be higher than that for people who have not disengaged). The number of suicide deaths per year is calculated as $\lambda a$, where $a$ is the suicide attempt rate and $\lambda$ is attempt lethality (i.e., the proportion of suicide attempts that are fatal, assumed to be constant). Self-harm hospitalisation and suicide death rate estimates derived from the system dynamics model and from HealthStats NSW (http://www.healthstats.nsw.gov.au/) are presented in figure S13.


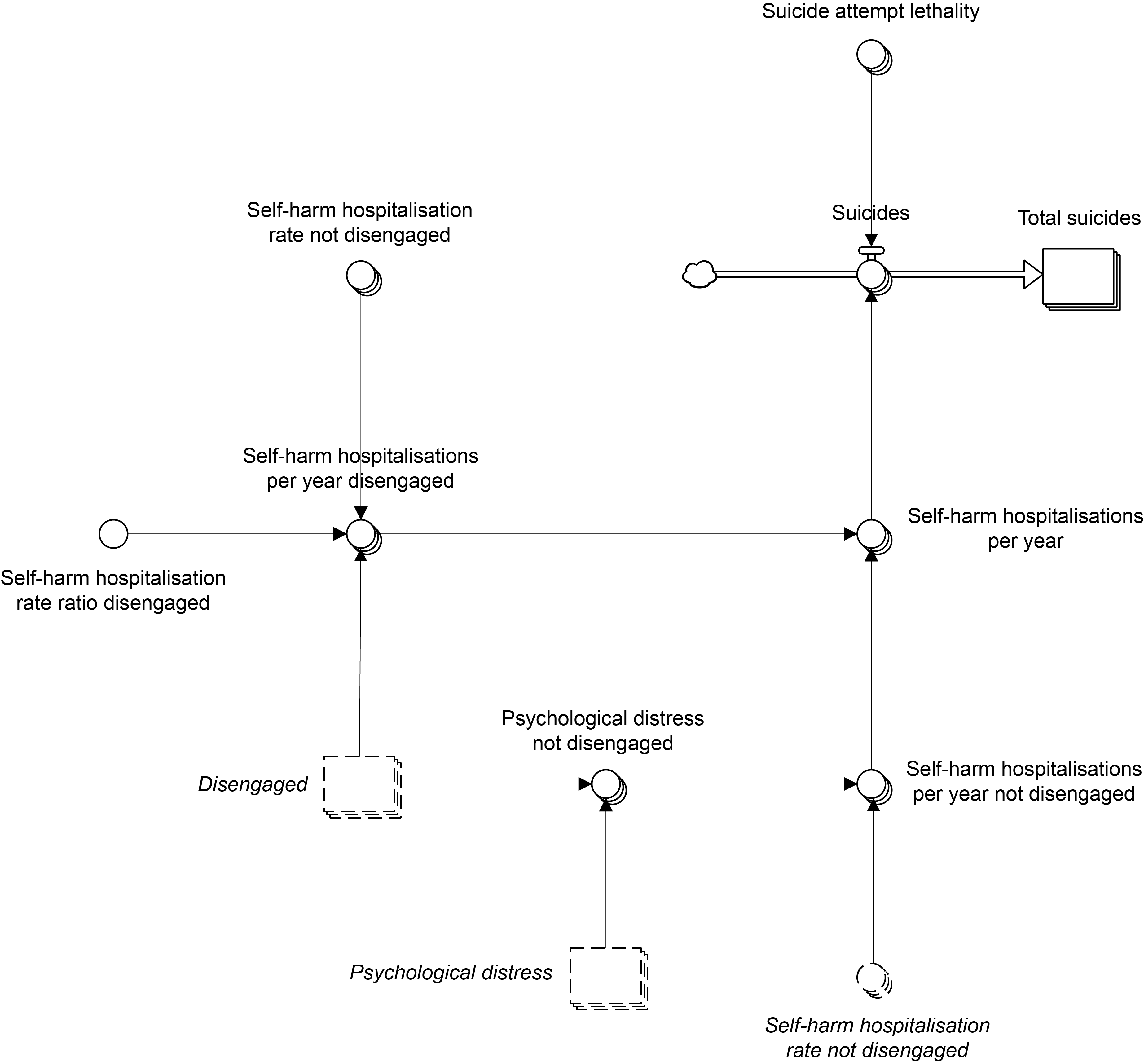


Figure S12. Structure of the suicidal behaviour sector.


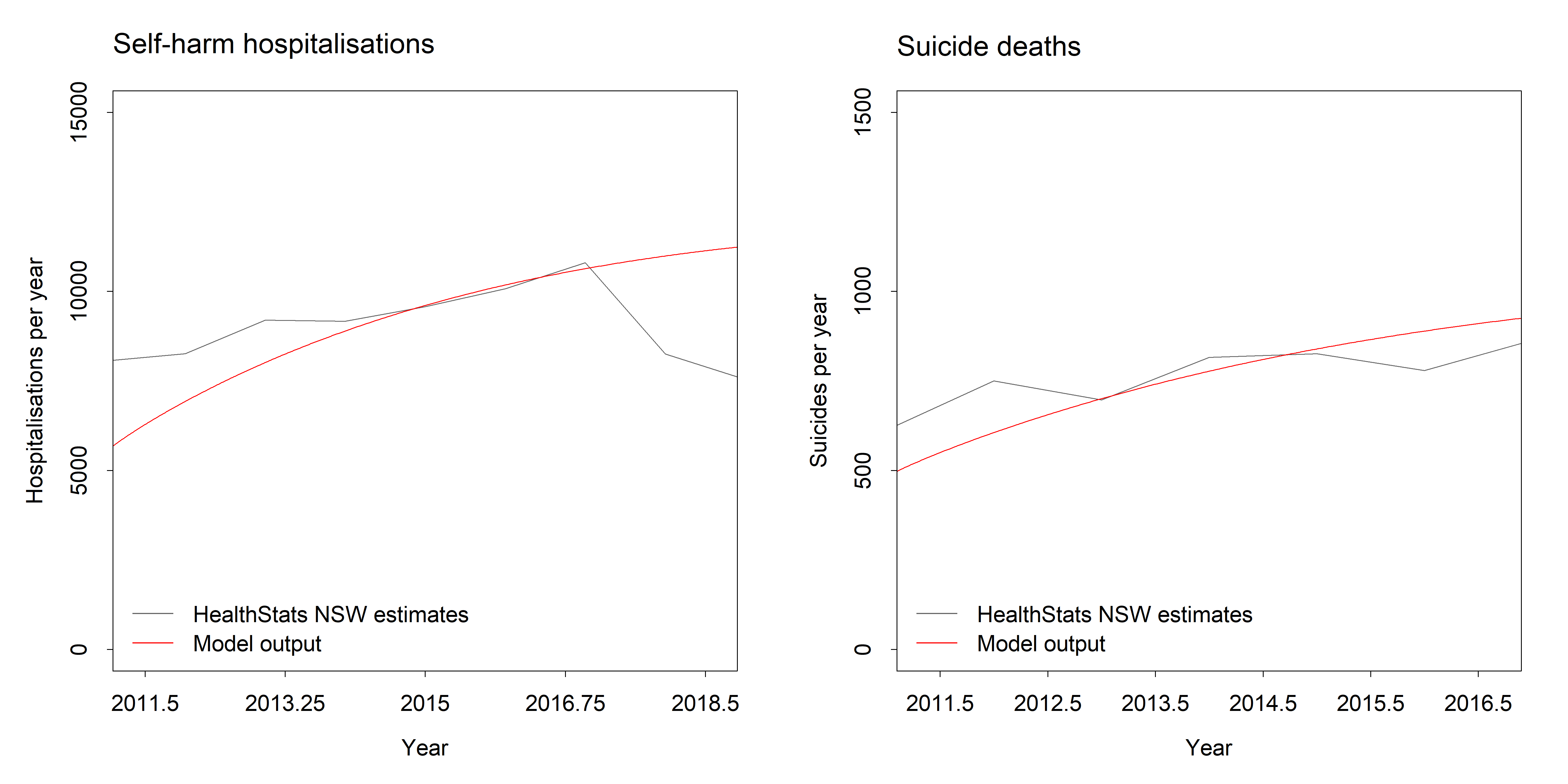


Figure S13. Self-harm hospitalisation and suicide death rate estimates (for all PHN catchments combined) derived from the system dynamics model and from HealthStats NSW (http://www.healthstats.nsw.gov.au/).

References

Australian Institute of Health and Welfare, 2018. Mental health services − in brief 2018. Cat. no. HSE 211. Australian Institute of Health and Welfare, Canberra.

Christensen, H., Griffiths, K. M., Jorm, A. F., 2004. Delivering interventions for depression by using the internet: randomised controlled trial. Br. Med. J. 328, 265.

Michael, T., Zetsche, U., Margraf, J., 2007. Epidemiology of anxiety disorders. Psychiatry 6, 136−142.

Russ, T. C., Stamatakis, E., Hamer, M., Starr, J. M., Kivimäki, M., Batty, G. D., 2012. Association between psychological distress and mortality: individual participant pooled analysis of 10 prospective cohort studies. Br. Med. J. 345, e4933.

Tyrer, P., Morgan, J., Van Horn, E., Jayakody, M., Evans, K., Brummell, R., White, T., Baldwin, D., Harrison-Read, P., Johnson, T., 1995. A randomised controlled study of close monitoring of vulnerable psychiatric patients. Lancet 345, 756−759.

Weissman, M. M., Bland, R. C., Canino, G. J., Faravelli, C., Greenwald, S., Hwu, H., Joyce, P. R., Karam, E. G., Lee, C., Lellouch, J., Lépine, J., Newman, S. C., Rubio-Stipec, M., Wells, J. E., Wickramaratne, P. J., Wittchen, H., Yeh, E., 1996. Cross-national epidemiology of major depression and bipolar disorder. J. Am. Med. Assoc. 276, 293−299.

3. Intervention definitions and parameter assumptions

Table S2. Intervention definitions and parameter assumptions. Parameters determining the direct effects of each intervention can be modified via an interactive model interface, enabling users to assess the impact of parameter assumptions on model outputs.

| Intervention | Description |
| --- | --- |
|  |  |
| 1. Post-suicide attempt care | Post-attempt care is an active outreach and enhanced contact program that aims to reduce readmission in those presenting to services after a suicide attempt. It includes individually tailored contact, solution focused counselling, and motivations to ensure adherence to follow-up treatments and continuity of contact.  Parameters determining the direct effects of this intervention are:  *Maximum post-attempt care rate* – the maximum proportion of patients hospitalised for a suicide attempt receiving post-attempt care. The default value (1) assumes that post-attempt care will be provided to all patients hospitalised for a suicide attempt (after an initial scale up period).  *Post-attempt care effect* – the proportion of potential repeat suicide attempts expected among patients receiving post-attempt care. The default value (0.398) implies that 39.8% of repeat attempts that would have occurred without post-attempt care actually occur when post-attempt care is provided; i.e., post-attempt care is assumed to prevent 60.2% of potential repeat suicide attempts. The default estimate is derived from Hvid et al. (2011, Nord. J. Psychiatry 65, 292-298).  *Effect duration (weeks)* – the average time in weeks after a suicide attempt that post-attempt care has an effect on the probability of a repeat attempt. The default value of 52.1 weeks implies that, on average, post-attempt care reduces the repeat self-harm rate for 1 year after an attempt. After this time, post-attempt care is assumed to have no impact on the suicide attempt rate.   *Repeat self-harm rate per year* – the probability that a person will self-harm in the year after a suicide attempt without post-attempt care. The default value (0.179) implies that 17.9% of people hospitalised for self-harm will re-attempt within 1 year (i.e., assuming they don't receive post-attempt care); this estimate is derived from Carroll et al. (2014, PLoS ONE 9, e89944). |
| 2. Safety planning | Safety planning aims to reduce suicidal behaviour through the provision of a specific plan for staying safe during crisis to suicidal patients presenting to an emergency department. The modelled intervention also includes up to 2 follow-up phone calls to monitor suicide risk and support treatment engagement (see Stanley et al., 2018, JAMA Psychiatry 75, 894-900).  Parameters determining the direct effects of this intervention are:  *Maximum rate per ED visit* – the maximum proportion of suicide-related emergency department presentations in which a safety plan is provided. The default value (0.7) assumes that a safety plan is provided to 70% of patients presenting to an emergency department for suicidal ideation or behaviour.  *Effect on self-harm rate* – the proportion of potential re-presentations for suicidal ideation or behaviour expected among patients provided with a safety plan. The default value (0.847) implies that 84.7% of suicide-related re-presentations that would have occurred without safety planning actually occur when a safety plan is provided; i.e., safety planning is assumed to prevent 15.3% of potential re-presentations for suicidal ideation or behaviour. The default value is derived from Miller et al. (2017, JAMA Psychiatry 74, 563-570).   *Effect duration (weeks)* – the average time in weeks after a suicide-related emergency department presentation that safety planning has an effect on the probability of re-presentations for suicidal ideation or behaviour (the default is 8 weeks).  *Re-presentation rate per year* – the expected number of re-presentations for suicidal ideation or behaviour in the year after an initial suicide-related emergency department attendance. The default value (3.84) is derived from Perera et al. (2018, Med. J. Aust. 208, 348-353), and implies that in the year after presenting to an emergency department for suicidal ideation or behaviour, patients will re-present 3.8 times (on average). |
| 3. Community-based acute care services | Responsive clinical mental health services delivered by community mental health teams. People in suicidal crisis may call and request either a home-based visit or a centre-based visit, depending on their level of functioning and risk.  Parameters determining the direct effects of this intervention are:  *Maximum self-referral rate* – the maximum proportion of people presenting to emergency departments for suicidal ideation or behaviour who would self-refer to community-based acute care services (i.e., if these were made available). The default value (0.7) assumes that 70% of people in suicidal crisis who would normally present to an emergency department would contact community-based services instead.  *Effect on self-harm rate* – the proportion of potential re-presentations for suicidal ideation or behaviour expected among patients referred to community-based acute care services. The default value (0.398) implies that 39.8% of re-presentations that would have occurred if a person in crisis was treated in an emergency department actually occur when community-based care is provided; i.e., community-based acute care is assumed to prevent 60.2% of potential re-presentations for suicidal ideation or behaviour. The default value is derived from Hvid et al. (2011, Nord. J. Psychiatry 65, 292-298). (Note that the default value is the estimated effect of post-attempt care on the repeat self-harm rate, but that the duration of effect of community-based acute care is assumed to be much shorter than that of post-attempt care.)  *Effect duration (weeks)* – the average time in weeks after referral to services that community-based acute care has an effect on the probability of repeat episodes of suicidal ideation or behaviour (the default value is 2 weeks).  *Re-presentation rate per year* – the expected number of re-presentations for suicidal ideation or behaviour in the year after an initial suicide-related emergency department attendance. The default value (3.84) is derived from Perera et al. (2018, Med. J. Aust. 208, 348-353), and implies that in the year after presenting to an emergency department for suicidal ideation or behaviour, patients will re-present 3.8 times (on average). |
| 4. Safe space services | Based on the United Kingdom’s Safe Haven café model, this intervention provides an alternative point of contact with mental health services for people experiencing acute psychological distress who may otherwise present to an emergency department.  Parameters determining the direct effects of this intervention are:  *Maximum self-referral rate* – the maximum proportion of people presenting to emergency departments for suicidal ideation or behaviour who would self-refer to a safe space alternative (i.e., if it were made available). The default value (0.7) assumes that 70% of people in suicidal crisis who would normally present to an emergency department would present to a safe space alternative instead.  *Effect on self-harm rate* – the proportion of potential re-presentations for suicidal ideation or behaviour expected among patients referred to a safe space service. The default value (0.398) implies that 39.8% of re-presentations that would have occurred if a person in suicidal crisis was treated in an emergency department actually occur when care is provided in a safe space alternative; i.e., care in a safe space alternative is assumed to prevent 60.2% of potential re-presentations for suicidal ideation or behaviour. The default value is derived from Hvid et al. (2011, Nord. J. Psychiatry 65, 292-298). (Note that the default value is the estimated effect of post-attempt care on the repeat self-harm rate, but that the duration of effect of safe space care is assumed to be much shorter than that of post-attempt care.)  *Effect duration (weeks)* – the average time in weeks that care provided in a safe space service has an effect on the probability of repeat episodes of suicidal ideation or behaviour (the default value is 2 weeks).  *Re-presentation rate per year* – the expected number of re-presentations for suicidal ideation or behaviour in the year after an initial suicide-related emergency department attendance. The default value (3.84) is derived from Perera et al. (2018, Med. J. Aust. 208, 348-353), and implies that in the year after presenting to an emergency department for suicidal ideation or behaviour, patients will re-present 3.8 times (on average). |
| 5. Technology-enabled care coordination | Technology-enabled care coordination involves the use of online technology to facilitate delivery of multidisciplinary team-based care, in which medical and allied health professionals consider all relevant treatment options and collaboratively develop an individual treatment and care plan for each patient.  Parameters determining the direct effects of this intervention are:  *Maximum rate per service* – the maximum proportion of mental health services provided that involve technology-enabled coordinated care. This proportion will depend on the number of medical and allied mental health professionals adopting online care coordination technologies, as well as the number of patients consenting to the use of these technologies in the management of their care (i.e., take-up among service providers and patients). The default value (0.7) assumes that when fully implemented, technology-enabled coordinated care will be provided in 70% of mental health services completed.  *Effect on recovery rate* – the multiplicative effect of technology-enabled coordinated care on the per-service recovery rate (i.e., the probability that a patient’s level of psychological distress will decrease after receiving treatment). The default estimate (1.177) is derived from Woltmann et al. (2012, Am. J. Psychiatry 169, 790-804), and implies that technology-enabled coordinated care increases the per-service probability of a reduction in psychological distress by 17.7%.  *Effect on referrals to specialised care* – the multiplicative effect of technology-enabled coordinated care on general practitioners’ rates of referral to specialised psychiatric care (i.e., psychiatrists and allied health services). The default value (1.266) implies that technology-enabled coordinated care increases the per-consultation probability that a general practitioner will refer a patient with high or very high psychological distress to specialised psychiatric care by 26.6%, and is derived from Badamgarav et al. (2003, Am. J. Psychiatry 160, 2080-2090).  *Effect on disengagement* – the multiplicative effect of technology-enabled coordinated care on rates of disengagement from mental health services (including disengagement while waiting for services and disengagement resulting from dissatisfaction with services received). The default estimate (0.520) is derived from Badamgarav et al. (2003, Am. J. Psychiatry 160, 2080-2090), and implies that technology-enabled coordinated care reduces rates of disengagement by 48.0%. |
| 6. Family psychoeducation and support | Provision of education and support to families and carers of patients presenting to or engaged with mental health services, with the aim of supporting family or carer involvement in the management of diagnosed mental disorders.  Parameters determining the direct effects of this intervention are:  *Maximum rate per patient* – the maximum proportion of patients with a chronic mental disorder who would consent to having their family involved in the management of their care. The default value (0.553) implies that family education and support would be provided to a maximum of 55.3% of patients with a chronic mental disorder, and is derived from Shimazu et al. (2011, Br. J Psychiatry 198, 385-390).  *Effect on recovery rate* – the multiplicative effect of family education and support on the recovery rate among patients with a chronic mental disorder treated by a GP, psychiatrist, or allied mental health professional. The default value (2.52) is derived from Shimazu et al. (2011, Br. J Psychiatry 198, 385-390), and implies that family education and support increase the per-service probability of recovery by a factor of 2.5. |
| 7. General practitioner (GP) training | Short (1-2 days) training programs aimed at reducing suicidal ideation through referral to specialised psychiatric services. This includes people who may be thinking about suicide for the first time or have survived a previous attempt.  Parameters determining the direct effects of this intervention are:  *Maximum training rate* – the maximum proportion of mental health-related GP services provided by GPs who have attended a training program (this value increases as the number of GPs attending training programs increases). The default value (0.7) implies that at most 70% of mental health-related GP services will be provided by a GP who has attended a training program.  *GP training effect* – the multiplicative effect of GP training on the rate of referral to psychiatrist and allied mental health services. The default value (1.4375) implies that GPs who have received training are 1.44 times more likely to refer patients with high or very high levels of psychological distress (Kessler 10 scores 22 and above) than a GP who has not received training. The default estimate is derived from Pfaff et al. (2001, Med. J. Aust. 174, 222-226). |
| 8. Awareness campaigns | Population-wide mental health education programs aimed at reducing stigma, improving recognition of suicide risk, and encouraging help-seeking. This intervention increases the per capita rates at which people perceive a need for mental health services and seek help from a general practitioner or online services.  Parameters determining the direct effects of this intervention are:  *Effect on help seeking* – the multiplicative effect of community-based education programs on the rate (per year) that a psychologically-distressed person not engaged with mental health services will perceive a need for treatment and the rate that a person perceiving a need for care will seek help from a GP or online services. The default value (1.585) is derived from Jorm et al. (2003, Psychol. Med. 33, 1071-1079), and assumes that awareness campaigns will increase the rate of help seeking for mental health problems by 58.5%. |
| 9. Social connectedness programs | Community support programs and services that increase social connectedness, reducing isolation and enhancing resilience in the face of adversity.  Parameters determining the direct effects of this intervention are:  *Sense of Community Index target* – the maximum Sense of Community Index (SCI) that could be achieved with the planned social connectedness program(s), where the SCI ranges from 0 to 12, with 12 corresponding to the highest possible sense of community (see Chipuer and Pretty, 1999, J. Community Psychol. 27, 643-658). The default value (9.61) corresponds to an increase in the SCI (relative to the baseline value, 9.15) of 5% (Handley et al., 2012, Soc. Psychiatry Psychiatr. Epidemiol. 47, 1281-1290).  *Effect on distress* – the multiplicative effect of a 1-unit increase in the SCI on distress onset rates. The default value (0.640) is derived from Handley et al. (2012, Soc. Psychiatry Psychiatr. Epidemiol. 47, 1281-1290), and implies that a 1-unit increase in the SCI reduces the rate at which people become psychologically distressed by 36.0%.  *Effect on attempt lethality* – the multiplicative effect of a 1-unit increase in the SCI on suicide attempt lethality. The default value (0.964) assumes that an increase in the SCI from its baseline value (9.15) to 12 would reduce suicide attempt lethality by 10%. |
| 10. GP services capacity increase | An increase in the annual rate of growth in general practitioner services capacity (i.e., the maximum number of services that can be provided per week) equal to 5% of capacity at the start of 2011. |
| 11. Psychiatrist and allied health services capacity increase | An increase in the annual rate of growth in psychiatrist and allied health services capacity (i.e., the maximum number of services that can be provided per week) equal to 5% of capacity at the start of 2011. |
| 12. Psychiatric hospital capacity increase | An increase in the annual rate of growth in public psychiatric hospital capacity (i.e., the maximum number of admissions per week) equal to 5% of capacity at the start of 2011. |
| 13. Community mental health care services capacity increase | An increase in the annual rate of growth in community mental health care services capacity (i.e., the maximum number of services that can be provided per week) equal to 5% of capacity at the start of 2011. |
|  |  |

4. Numerical inputs and data sources

Table S3. Numerical inputs and data sources. Inputs highlighted in red were varied in the sensitivity analyses (see Methods section of the paper).

| Input label | Value(s) | Notes |
| --- | --- | --- |
|  |  |  |
| Population sector |  |  |
|  |  |  |
| Birth rate increase per year | -0.0222711641111 (Cen E Syd), -0.0111501969335 (N Syd), -0.0182508884959 (W Syd), -0.0102061137698 (Nepean BM), -0.00442036941355 (SW Syd), -0.0087291326701 (SE NSW), -0.00200859522954 (W NSW), -0.0160983362356 (HNECC), -0.0312589834253 (N Coast), -0.00721805511141 (Murrumbidgee) | Fractional rate of increase per year. Estimated via constrained optimisation |
| Birth rate per year initial | 0.014091971 (Cen E Syd), 0.012301082 (N Syd), 0.01714743 (W Syd), 0.014232812 (Nepean BM), 0.014651325 (SW Syd), 0.010199086 (SE NSW), 0.013000951 (W NSW), 0.012718379 (HNECC), 0.010573347 (N Coast), 0.010340173 (Murrumbidgee) | Per capita birth rate per year. Estimated via constrained optimisation. |
| Death rate increase per year | -0.0114322546459 (Cen E Syd), -0.0137442690807 (N Syd), -0.0101238079884 (W Syd), 0.0201820314502 (Nepean BM), 0.0028178446167 (SW Syd), 0.0129791651441 (SE NSW), 0.000425456638915 (W NSW), 0.00815873905427 (HNECC), 0.00482397158981 (N Coast), 0.00848551183661 (Murrumbidgee) | Fractional rate of increase per year. Estimated via constrained optimisation |
| Death rate per year initial | 0.005826676 (Cen E Syd), 0.006451182 (N Syd), 0.004923075 (W Syd), 0.005695421 (Nepean BM), 0.005488966 (SW Syd), 0.008127316 (SE NSW), 0.008905438 (W NSW), 0.00876396 (HNECC), 0.009915163 (N Coast), 0.008659258 (Murrumbidgee) | Per capita mortality rate per year. Estimated via constrained optimisation. |
| Emigration rate | 0.0164048013637 (Cen E Syd), 0.0189060349234 (N Syd), 0.0121248034052 (W Syd), 0.0149141111855 (Nepean BM), 0.0178232809532 (SW Syd), 0.0126447195378 (SE NSW), 0.0139944480059 (W NSW), 0.0140276374442 (HNECC), 0.0170161503448 (N Coast), 0.0149933179854 (Murrumbidgee) | Per capita emigration rate per year. Estimated via constrained optimisation. |
| Immigration rate | 754.937078633 (Cen E Syd), 449.676955037 (N Syd), 432.331113784 (W Syd), 129.409741397 (Nepean BM), 496.140169176 (SW Syd), 242.924204154 (SE NSW), 82.7667519725 (W NSW), 484.19377441 (HNECC), 248.617138243 (N Coast), 81.7366648166 (Murrumbidgee) | Number of people immigrating per week. Estimated via constrained optimisation. |
|  |  |  |
| Psychological distress sector |  |  |
|  |  |  |
| Death rate ratio psychological distress | 1.27 | Derived from Russ et al. (2012, Br. Med. J. 345, e4933) |
| Pre-intervention psychological distress onset rate | 0.119011494861 (Cen E Syd), 0.108388793297 (N Syd), 0.112259208824 (W Syd), 0.124884593428 (Nepean BM), 0.111105344927 (SW Syd), 0.114130743421 (SE NSW), 0.0920990326748 (W NSW), 0.117876816185 (HNECC), 0.134206379675 (N Coast), 0.0816813507822 (Murrumbidgee) | Per capita rate per year. Estimated via constrained optimisation. |
| Psychological distress prevalence initial | 0.177152458116 (Cen E Syd), 0.168084759793 (N Syd), 0.194337119769 (W Syd), 0.180065155934 (Nepean BM), 0.191306612928 (SW Syd), 0.17625455125 (SE NSW), 0.179896944552 (W NSW), 0.157313505126 (HNECC), 0.181142770755 (N Coast), 0.186352997238 (Murrumbidgee) | Estimated via constrained optimisation |
| Recovery base rate | 0.06833333 | Per capita spontaneous recovery rate per year. Derived from Jokela et al. (2011, J. Affect. Disord. 130, 454−461). |
|  |  |  |
| Mental health services sector |  |  |
|  |  |  |
| Additional admissions rate non-specialised hospital care | 0.172237023678 (Cen E Syd), 0.210489892679 (N Syd), 0.125910510877 (W Syd), 0.146600477178 (Nepean BM), 0.0660082636623 (SW Syd), 0.0735351034059 (SE NSW), 0.0829264563755 (W NSW), 0.141633877616 (HNECC), 0.0724701600496 (N Coast), 0.0456652301032 (Murrumbidgee) | Per capita rate per year. Estimated via constrained optimisation. |
| Additional CMHC service contacts rate | 9.18283853997 (Cen E Syd), 13.1401313274 (N Syd), 4.3682283505 (W Syd), 4.81128259552 (Nepean BM), 5.80267702111 (SW Syd), 6.85884037758 (SE NSW), 11.8590568858 (W NSW), 11.4354503597 (HNECC), 7.71889719303 (N Coast), 4.48789397743 (Murrumbidgee) | Per capita rate per year. Estimated via constrained optimisation. |
| Additional psychiatrist and allied services rate | 9.56865410821 (Cen E Syd), 10.4277119905 (N Syd), 5.99840502791 (W Syd), 5.93232976862 (Nepean BM), 3.82234625728 (SW Syd), 5.46023684983 (SE NSW), 3.67941057627 (W NSW), 5.42560626529 (HNECC), 6.59605687269 (N Coast), 1.52712782673 (Murrumbidgee) | Per capita rate per year. Estimated via constrained optimisation. |
| CMHC services capacity increase per year | 814.976621108 (Cen E Syd), 509.889324369 (N Syd), 72.244112476 (W Syd), 0 (Nepean BM), 0 (SW Syd), 29.747844652 (SE NSW), 0 (W NSW), 0 (HNECC), 0 (N Coast), 0 (Murrumbidgee) | Annual increase in the total number of community mental health care service contacts per week. Default values were derived from service usage data for 2014–2018 published by the Australian Institute of Health and Welfare (available at: https://www.aihw.gov.au/reports-data/health-welfare-services/mental-health-services/data). |
| CMHC services capacity initial | 6165.82703386 (Cen E Syd), 5885.96517365 (N Syd), 3490.42930896 (W Syd), 1806.94082872 (Nepean BM), 6135.16348998 (SW Syd), 4121.36003957 (SE NSW), 3414.63877971 (W NSW), 13006.9811907 (HNECC), 4434.73786451 (N Coast), 1490.79455675 (Murrumbidgee) | The initial number of community mental health care service contacts per week (i.e., at the start of 2011). Estimated via constrained optimisation. |
| CMHC services referral rate ED | 0.491095419 | Fraction of patients discharged from emergency department care referred to community mental health care services. Estimated via constrained optimisation. |
| Disengaged to perceived need for services rate | 3 | Per capita rate per year. Default value assumes patients who have disengaged from services consider re-engaging after 4 months (on average). |
| ED presentation rate | 0.00949508851381 (Cen E Syd), 0.00812102352266 (N Syd), 0.00685301981251 (W Syd), 0.0125542791195 (Nepean BM), 0.00959524724231 (SW Syd), 0.0129621164035 (SE NSW), 0.035020179663 (W NSW), 0.0139429143795 (HNECC), 0.0148125711676 (N Coast), 0.0384040827402 (Murrumbidgee) | Per capita rate per year. Estimated via constrained optimisation. |
| ED presentation rate ratio perceived need for services | 6.704169646 | Estimated via constrained optimisation |
| GP services capacity increase per year | 239.525438597 (Cen E Syd), 122.488801907 (N Syd), 144.897866471 (W Syd), 81.0275429933 (Nepean BM), 156.916212662 (SW Syd), 187.932291281 (SE NSW), 64.5220026256 (W NSW), 334.097108028 (HNECC), 137.858781922 (N Coast), 38.7165712979 (Murrumbidgee) | Annual increase in the total number of mental health-related general practitioner consultations that can be completed per week. The default value was estimated using Medicare Benefits Schedule (MBS) data for 2012–2017 assuming services were operating at (near-) maximum capacity over this period. |
| GP services capacity initial | 2555.6320781 (Cen E Syd), 1075.76924122 (N Syd), 1675.82002194 (W Syd), 709.032268144 (Nepean BM), 1312.06816715 (SW Syd), 917.092995368 (SE NSW), 440.015486211 (W NSW), 2003.13141389 (HNECC), 1222.36946926 (N Coast), 291.836048946 (Murrumbidgee) | The initial number of mental health-related general practitioner consultations completed per week (i.e., at the start of 2011). Estimated via constrained optimisation. |
| Mean treatment duration non-specialised hospital care (weeks) | 0.8746473 | Derived from national data on mental health-related hospitalisations published by the Australian Institute of Health and Welfare (available at: https://www.aihw.gov.au/reports-data/health-welfare-services/mental-health-services/data) |
| Mean treatment duration online services (weeks) | 6 | Derived from Christensen et al. (2004, Br. Med. J. 328, 265) |
| Mean treatment duration psychiatric hospital (weeks) | 3.182598 | Derived from national data on mental health-related hospitalisations published by the Australian Institute of Health and Welfare (available at: https://www.aihw.gov.au/reports-data/health-welfare-services/mental-health-services/data) |
| Non-specialised hospital capacity increase per year | 7.89721617292 (Cen E Syd), 0 (N Syd), 1.1768725941 (W Syd), 3.32499611951 (Nepean BM), 3.99459694625 (SW Syd), 2.92222154587 (SE NSW), 0.545163014924 (W NSW), 2.12332606833 (HNECC), 4.27980207637 (N Coast), 0.487836227539 (Murrumbidgee) | Annual increase in the maximum number of general hospital admissions for mental disorders per week. The default value was estimated using hospital separations data for 2011–2018 available from HealthStats NSW (http://www.healthstats.nsw.gov.au) and data on the provision of non-specialised mental health care in public hospitals published by the Australian Institute of Health and Welfare (available at: https://www.aihw.gov.au/reports-data/health-welfare-services/mental-health-services/data). |
| Non-specialised hospital capacity initial | 167.687534184 (Cen E Syd), 124.366126546 (N Syd), 100.665400543 (W Syd), 39.0789065488 (Nepean BM), 56.7988509289 (SW Syd), 36.5079654046 (SE NSW), 23.0043411314 (W NSW), 142.345306514 (HNECC), 23.3364659802 (N Coast), 12.8648708902 (Murrumbidgee) | The initial number of general hospital admissions for mental disorders per week (i.e., at the start of 2011). Estimated via constrained optimisation. |
| Perceiving need for services base rate | 0.375699223363 (Cen E Syd), 0.406248121713 (N Syd), 0.296647964481 (W Syd), 0.309317046965 (Nepean BM), 0.284454919892 (SW Syd), 0.321497916445 (SE NSW), 0.304483308858 (W NSW), 0.331825623082 (HNECC), 0.376216728878 (N Coast), 0.216206600583 (Murrumbidgee) | Per capita rate per year. Estimated via constrained optimisation. |
| Perceiving need for services rate increase per year | 0 | Estimated via constrained optimisation. Note that the estimated value entails that the per capita rate at which psychologically distressed people perceive a need for care remains constant over the simulation period. |
| Post-discharge CMHC services referral rate increase per year | 0.085308257 | Estimated via constrained optimisation using data on key performance indicators for Australian public mental health services published by the Australian Institute of Health and Welfare (available at: https://www.aihw.gov.au/reports-data/health-welfare-services/mental-health-services/data) |
| Post-discharge CMHC services referral rate initial | 0.528308732 | Fraction of patients discharged from hospital inpatient care referred to community mental health care services. Estimated via constrained optimisation using data on key performance indicators for Australian public mental health services published by the Australian Institute of Health and Welfare (available at: https://www.aihw.gov.au/reports-data/health-welfare-services/mental-health-services/data). |
| Post-discharge non-CMHC services referral proportion GP | 0.557650176 | Proportion of patients not referred to community mental health care services after discharge from hospital inpatient care referred to a general practitioner. Estimated via constrained optimisation. Note that patients not referred to community mental health care services or a general practitioner are referred to psychiatrist and allied health services. |
| Pre-intervention disengagement rate hospital care | 0.04779412 | Derived from state-level consumer survey data for 2017-18 published by the Australian Institute of Health and Welfare (available at: https://www.aihw.gov.au/reports-data/health-welfare-services/mental-health-services/data) |
| Pre-intervention disengagement rate non-hospital care | 0.04459478 | Derived from state-level consumer survey data for 2017-18 published by the Australian Institute of Health and Welfare (available at: https://www.aihw.gov.au/reports-data/health-welfare-services/mental-health-services/data) |
| Pre-intervention disengagement rate waiting | 0.2620284 | Per capita rate per year. Derived from Tyrer et al. (1995, Lancet 345, 756−759) |
| Pre-intervention seeking help online services rate | 0.15 | Per capita rate per year. Derived from Australian Bureau of Statistics (2012, Information paper. Use of the Kessler psychological distress scale in ABS health surveys, Australia, 2007-08. Cat. no. 4817.0.55.001. Australian Bureau of Statistics, Canberra). |
| Psychiatric hospital admission proportion | 0.862489067 | Estimated via constrained optimisation |
| Psychiatric hospital capacity increase per year | 12.8435343821 (Cen E Syd), 0 (N Syd), 3.92438290314 (W Syd), 6.12137224526 (Nepean BM), 7.0014973743 (SW Syd), 4.6454286718 (SE NSW), 0.915503699016 (W NSW), 5.38278431673 (HNECC), 6.39468347364 (N Coast), 0.941448980035 (Murrumbidgee) | Annual increase in the maximum number of psychiatric hospital admissions per week. The default value was estimated using hospital separations data for 2011–2018 available from HealthStats NSW (http://www.healthstats.nsw.gov.au) and data on the provision of specialised psychiatric care in public hospitals published by the Australian Institute of Health and Welfare (available at: https://www.aihw.gov.au/reports-data/health-welfare-services/mental-health-services/data). |
| Psychiatric hospital capacity initial | 226.400646444 (Cen E Syd), 174.850020534 (N Syd), 131.702011792 (W Syd), 48.1714938085 (Nepean BM), 74.6479204979 (SW Syd), 48.5746836305 (SE NSW), 31.5612461435 (W NSW), 188.367930164 (HNECC), 31.4761154194 (N Coast), 16.8113788199 (Murrumbidgee) | The initial number of psychiatric hospital admissions per week (i.e., at the start of 2011). Estimated via constrained optimisation. |
| Psychiatrist and allied services capacity increase per year | 275.304151491 (Cen E Syd), 150.91606852 (N Syd), 127.821627065 (W Syd), 88.6767086012 (Nepean BM), 173.377024779 (SW Syd), 249.78070476 (SE NSW), 92.9806676707 (W NSW), 464.672654604 (HNECC), 259.523783336 (N Coast), 48.1152170358 (Murrumbidgee) | Annual increase in the total number of psychiatrist and allied services that can be provided per week. The default value was estimated using MBS data for 2012–2017 assuming services were operating at (near-) maximum capacity over this period. |
| Psychiatrist and allied services capacity initial | 8995.02091937 (Cen E Syd), 5258.83082066 (N Syd), 4286.06248639 (W Syd), 1589.18453667 (Nepean BM), 3197.79503759 (SW Syd), 2220.55727895 (SE NSW), 700.2883739 (W NSW), 3906.55296517 (HNECC), 2229.44896938 (N Coast), 313.580193333 (Murrumbidgee) | The initial number of psychiatrist and allied health services provided per week (i.e., at the start of 2011). Estimated via constrained optimisation. |
| Psychological treatment rate GP services | 0.4818195 | Derived from national data on mental health-related general practitioner services published by the Australian Institute of Health and Welfare (available at: https://www.aihw.gov.au/reports-data/health-welfare-services/mental-health-services/data) |
| Recovery base rate CMHC services | 0.02274479 | Per-service recovery rate derived from data on patient outcomes and numbers of services per patient per year published online by the Australian Institute of Health and Welfare (https://www.aihw.gov.au/reports-data/health-welfare-services/mental-health-services/data) |
| Recovery rate mental health treatment | 0.08917693 | Per-service recovery rates derived from treatment effectiveness estimates reported in Thase et al. (1997, Arch. Gen. Psychiatry 54, 1009−1015) and data on numbers of services per patient per year published online by the Australian Institute of Health and Welfare (https://www.aihw.gov.au/reports/primary-health-care/medicare-subsidised-gp-allied-health-and-specialis/data). |
| Recovery rate online services | 0.4 | Derived from Christensen et al. (2004, Br. Med. J. 328, 265) and Cuijpers et al. (2009, Br. J. Gen. Pract., doi: 10.3399/bjgp09X395139) |
| Recovery rate ratio GP services | 0.7243705 | Derived from Cuijpers et al. (2009, Br. J. Gen. Pract., doi: 10.3399/bjgp09X395139) |
| Recovery rate specialised hospital care | 0.3712737 | Derived from Thase et al. (1997, Arch. Gen. Psychiatry 54, 1009−1015) |
| Referral rate CMHC services GP | 0.003907 | Derived from national data on mental health-related general practitioner services published by the Australian Institute of Health and Welfare (available at: https://www.aihw.gov.au/reports-data/health-welfare-services/mental-health-services/data) |
| Referral rate online services | 0.047724167 | Derived from national data on mental health-related general practitioner services published by the Australian Institute of Health and Welfare (available at: https://www.aihw.gov.au/reports-data/health-welfare-services/mental-health-services/data) |
| Referral rate psychiatrist and allied services increase per year | 0.008541323 | Estimated via constrained optimisation |
| Referral rate psychiatrist or allied services initial | 0.077617522 | Initial fraction of patients consulting a general practitioner referred to psychiatrist and allied health services (i.e., at the start of 2011). Estimated via constrained optimisation using national data on mental health-related general practitioner services published by the Australian Institute of Health and Welfare (available at: https://www.aihw.gov.au/reports-data/health-welfare-services/mental-health-services/data). |
| Referred to psychiatric hospital rate | 0.0225711063414 (Cen E Syd), 0.0281097130194 (N Syd), 0.0269883583381 (W Syd), 0.0269809091258 (Nepean BM), 0.0169836672718 (SW Syd), 0.0114913285081 (SE NSW), 0.00243977184107 (W NSW), 0.0268235837054 (HNECC), 0.00685774170101 (N Coast), 0.00320785501756 (Murrumbidgee) | Fraction of patients attending an appointment with a psychiatrist or allied health services referred to psychiatric hospital care. Estimated via constrained optimisation. |
| Seeking help GP services base rate | 2.77573657166 (Cen E Syd), 2.2069526767 (N Syd), 2.40040266192 (W Syd), 2.69630341549 (Nepean BM), 1.62296563791 (SW Syd), 2.42342728725 (SE NSW), 2.1609793249 (W NSW), 2.76393069473 (HNECC), 3.182285833 (N Coast), 1.27613451923 (Murrumbidgee) | Per capita rate per year. Estimated via constrained optimisation. |
| Seeking help GP services rate increase per year | 0.037170406 | Default value assumes a yearly increase in the per capita help-seeking rate for each PHN catchment equal to 3.72% of the initial base rate (specified by the parameter *Seeking help GP services base rate*). Estimated via constrained optimisation. |
| Total hospital admission rate | 0.316295146 | Fraction of patients presenting to an emergency department for a mental health-related problem admitted to hospital. Derived from state-level data on mental health-related emergency department presentations published by the Australian Institute of Health and Welfare (available at: https://www.aihw.gov.au/reports-data/health-welfare-services/mental-health-services/data). |
|  |  |  |
| Suicidal behaviour sector |  |  |
|  |  |  |
| Self-harm hospitalisation rate not disengaged | 0.00348508323994 (Cen E Syd), 0.00349714461501 (N Syd), 0.00303788888107 (W Syd), 0.00437402649166 (Nepean BM), 0.00424175196738 (SW Syd), 0.00520340482377 (SE NSW), 0.00441267638157 (W NSW), 0.00547427995825 (HNECC), 0.00628895100871 (N Coast), 0.00645170691378 (Murrumbidgee) | Per capita rate per year. Estimated via constrained optimisation. |
| Self-harm rate ratio disengaged | 1.286165 | Derived from Australian Bureau of Statistics (2012, Information paper. Use of the Kessler psychological distress scale in ABS health surveys, Australia, 2007-08. Cat. no. 4817.0.55.001. Australian Bureau of Statistics, Canberra) |
| Suicide attempt lethality | 0.0961527819519 (Cen E Syd), 0.0961527819519 (N Syd), 0.0861630781015 (W Syd), 0.0896339751833 (Nepean BM), 0.0667618891233 (SW Syd), 0.0830185477171 (SE NSW), 0.109426295236 (W NSW), 0.0867307443653 (HNECC), 0.0798230115525 (N Coast), 0.0836119252559 (Murrumbidgee) | Estimated via constrained optimisation |
|  |  |  |
| Interventions |  |  |
|  |  |  |
| Acute care services effect duration (weeks) | 2 | Default value assumes community-based acute care reduces the probability of repeat episodes of suicidal ideation or behaviour for a mean of 2 weeks after referral |
| Acute care services effect on self-harm rate | 0.3975155 | Derived from Hvid et al. (2011, Nord. J. Psychiatry 65, 292−298) |
| Additional CMHC service contacts per patient (post-attempt care) | 18.24816 | Difference between the mean number of community mental health care service contacts per patient for patients receiving medium to longer term treatment and the mean number of service contacts per patient for all patients. Estimated using data published by the Australian Institute of Health and Welfare (2018, Mental health services − in brief 2018. Cat. no. HSE 211. Australian Institute of Health and Welfare, Canberra). |
| Assertive aftercare effect duration (weeks) | 52.14285714 | Default value assumes assertive aftercare reduces the probability of repeat self-harm for a mean of 52.1 weeks after a suicide attempt |
| Assertive aftercare effect estimate | 0.3975155 | Derived from Hvid et al. (2011, Nord. J. Psychiatry 65, 292−298) |
| Assertive aftercare treatment duration (weeks) | 13.14286 | Default value assumes post-attempt care is provided for a mean of 92 days after a suicide attempt. This is the minimum duration of medium to longer term community-based mental health treatment, as defined in Australian Institute of Health and Welfare (2018, Mental health services − in brief 2018. Cat. no. HSE 211. Australian Institute of Health and Welfare, Canberra) |
| ED referral rate acute care services | 0.4815356 | Proportion of patients presenting to an emergency department for suicidal ideation or behaviour admitted to hospital (patients requiring inpatient care are assumed to be admitted via an emergency department). Estimate derived from Perera et al. (2018, Med. J. Aust. 208, 348−353). |
| ED referral rate safe space services | 0.4815356 | Fraction of patients attending a safe space service referred to an emergency department. Default value is an estimate of the proportion of suicide-related emergency department presentations resulting in hospital admission (derived from Perera et al., 2018, Med. J. Aust. 208, 348−353) |
| Effect of awareness campaigns on mental health service engagement | 1.585327 | Derived from Jorm et al. (2003, Psychol. Med. 33, 1071-1079) |
| Effect of Sense of Community Index increase on awareness campaign effectiveness | 1.034002 | Assumes an increase in the Sense of Community Index from its baseline value (9.15) to the highest possible value (12) would increase the effect of awareness campaigns on the rate of help seeking by 10% |
| Effect of Sense of Community Index increase on distress | 0.64 | Derived from Handley et al. (2012, Soc. Psychiatry Psychiatr. Epidemiol. 47, 1281−1290) |
| Effect of Sense of Community Index increase on lethality | 0.9637119 | Default value assumes an increase in the Sense of Community Index from its baseline value (9.15) to 12 (the maximum possible value) would reduce suicide attempt lethality by 10% |
| Effect of technology-enabled care on disengagement | 0.5204988 | Derived from Badamgarav et al. (2003, Am. J. Psychiatry 160, 2080−2090) |
| Effect of technology-enabled care on recovery rate | 1.177321 | Derived from Woltmann et al. (2012, Am. J. Psychiatry 169, 790−804) |
| Effect of technology-enabled care on referrals to specialised care | 1.265913 | Derived from Badamgarav et al. (2003, Am. J. Psychiatry 160, 2080−2090) |
| Family psychoeducation effect estimate | 2.515152 | Derived from Shimazu et al. (2011, Br. J Psychiatry 198, 385-390) |
| GP training effect estimate | 1.4375 | Derived from Pfaff et al. (2001, Med. J. Aust. 174, 222-226) |
| Maximum acute care services referral rate | 0.7 | Default value assumes that a maximum of 70% of people in suicidal crisis who would usually present to an emergency department will contact community-based acute care services instead |
| Maximum acute care services referral rate safe space services | 0.3044022 | Fraction of patients attending a safe space service referred to community-based acute care services. Default value is an estimate of the proportion of suicide-related emergency department presentations that are potentially life-threatening but do not result in hospital admission (derived from Perera et al., 2018, Med. J. Aust. 208, 348−353). |
| Maximum assertive aftercare rate | 1 | Default value assumes that post-attempt care will be provided to all patients hospitalised for a suicide attempt when fully implemented |
| Maximum family psychoeducation rate | 0.5533981 | Derived from Shimazu et al. (2011, Br. J Psychiatry 198, 385-390) |
| Maximum GP training rate | 0.7 | Default value assumes that at most 70% of mental health-related GP services will be provided by a GP who has attended a training program |
| Maximum safe space services referral rate | 0.7 | Default value assumes that 70% of people in suicidal crisis who would normally present to an emergency department would present to a safe space alternative instead |
| Maximum safety plan rate | 0.7 | Default value assumes that a safety plan is provided to a maximum of 70% of patients presenting to an emergency department for suicidal ideation or behaviour |
| Maximum technology-enabled care rate per service | 0.7 | Default value assumes that technology-enabled coordinated care will be provided in 70% of mental health service contacts when fully implemented |
| Proportion high or very high distress engaged with services | 0.512977099 | Derived from Australian Bureau of Statistics (2012, Information paper. Use of the Kessler psychological distress scale in ABS health surveys, Australia, 2007-08. Cat. no. 4817.0.55.001. Australian Bureau of Statistics, Canberra) |
| Proportion of ED presentations involving paramedics or police | 0.224 | Derived from Australian Institute of Health and Welfare (2018, Mental health services − in brief 2018. Cat. no. HSE 211. Australian Institute of Health and Welfare, Canberra) |
| Proportion of patients with a diagnosed affective disorder | 0.2343458 | Derived from Australian Bureau of Statistics (2012, Information paper. Use of the Kessler psychological distress scale in ABS health surveys, Australia, 2007-08. Cat. no. 4817.0.55.001. Australian Bureau of Statistics, Canberra) |
| Repeat self-harm rate per year | 0.179 | Derived from Carroll et al. (2014, PLoS ONE 9, e89944) |
| Safe space services effect duration (weeks) | 2 | Default value assumes care provided in a safe space service reduces the probability of repeat episodes of suicidal behaviour or ideation for a mean of 2 weeks |
| Safe space services effect estimate | 0.3975155 | Derived from Hvid et al. (2011, Nord. J. Psychiatry 65, 292-298) |
| Safety planning effect duration (weeks) | 8 | Default value assumes safety planning reduces the probability of re-presentation for suicidal behaviour or ideation for a mean of 8 weeks after an initial suicide-related emergency department presentation |
| Safety planning effect estimate | 0.8474415 | Derived from Miller et al. (2017, JAMA Psychiatry 74, 563-570) |
| Self-harm re-presentation rate per year | 3.837889 | Derived from Perera et al. (2018, Med. J. Aust. 208, 348−353) |
| Sense of Community Index initial | 9.149557522 | Derived from Handley et al. (2012, Soc. Psychiatry Psychiatr. Epidemiol. 47, 1281−1290) |
| Sense of Community Index target | 9.607035 | Default value corresponds to a 5% increase in the Sense of Community Index (relative to the baseline value, 9.15) |
| Suicide-related ED presentation admission rate | 0.4815356 | Derived from Perera et al. (2018, Med. J. Aust. 208, 348−353) |
| Suicide-related ED presentation rate | 0.173505926 | Derived from Perera et al. (2018, Med. J. Aust. 208, 348−353) |
| Years to implement awareness campaigns | 2 | Default value assumes it will take 2 years to scale up awareness campaigns |
| Years to implement GP training | 3 | Default value assumes it will take 3 years to fully implement a GP training program |
| Years to implement technology-enabled care | 2 | Default value assumes it will take 2 years to fully implement technology-enabled coordinated care |
| Years to reach maximum acute care services rate | 2 | Default value assumes it will take 2 years to reach the maximum acute care services referral rate |
| Years to reach maximum family psychoeducation rate | 2 | Default value assumes it will take 2 years to reach the maximum family psychoeducation and support rate |
| Years to reach maximum safe space services referral rate | 2 | Default value assumes it will take 2 years to reach the maximum safe space services referral rate |
| Years to reach maximum safety plan rate | 2 | Default value assumes it will take 2 years to reach the maximum safety plan rate |
| Years to reach Sense of Community Index target | 10 | Default value assumes it will take 10 years to reach the Sense of Community Index target |
| Years to scale up assertive aftercare | 2 | Default value assumes it will take 2 years to fully implement an post-attempt care program |
|  |  |  |

5. Preliminary modelling of the impact of COVID-19

The substantial adverse mental health impacts of social dislocation and job loss resulting from the continuing COVID-19 pandemic (e.g., Moreno et al., 2020; Atkinson et al., 2020) were modelled as an abrupt increase in psychological distress incidence from 1 March 2020 that declines gradually until the end of the simulation period (the start of 2031; figure S14). Parameters controlling the simulated effect of the COVID-19 pandemic on psychological distress onset (see Table S4) were set to generate a maximum increase in psychological distress prevalence of 8.4% (in mid-2022), which results in a 6.5% increase in the total number of self-harm hospitalisations over the period 2020−2025, consistent with national-level modelling of the impacts of the pandemic on mental health outcomes (see Occhipinti et al., 2021). Figure S15 shows percentage reductions in cumulative numbers of suicides corresponding to those in figure 1 of the paper obtained when the impact of COVID-19 is included in the model projections. Although our approach to modelling the effects of COVID-related social dislocation and unemployment on psychological distress is relatively simple, the similarity between the results in figure 1 and figure S15 indicates that more detailed modelling is unlikely to significantly affect the principal conclusions drawn in the paper.


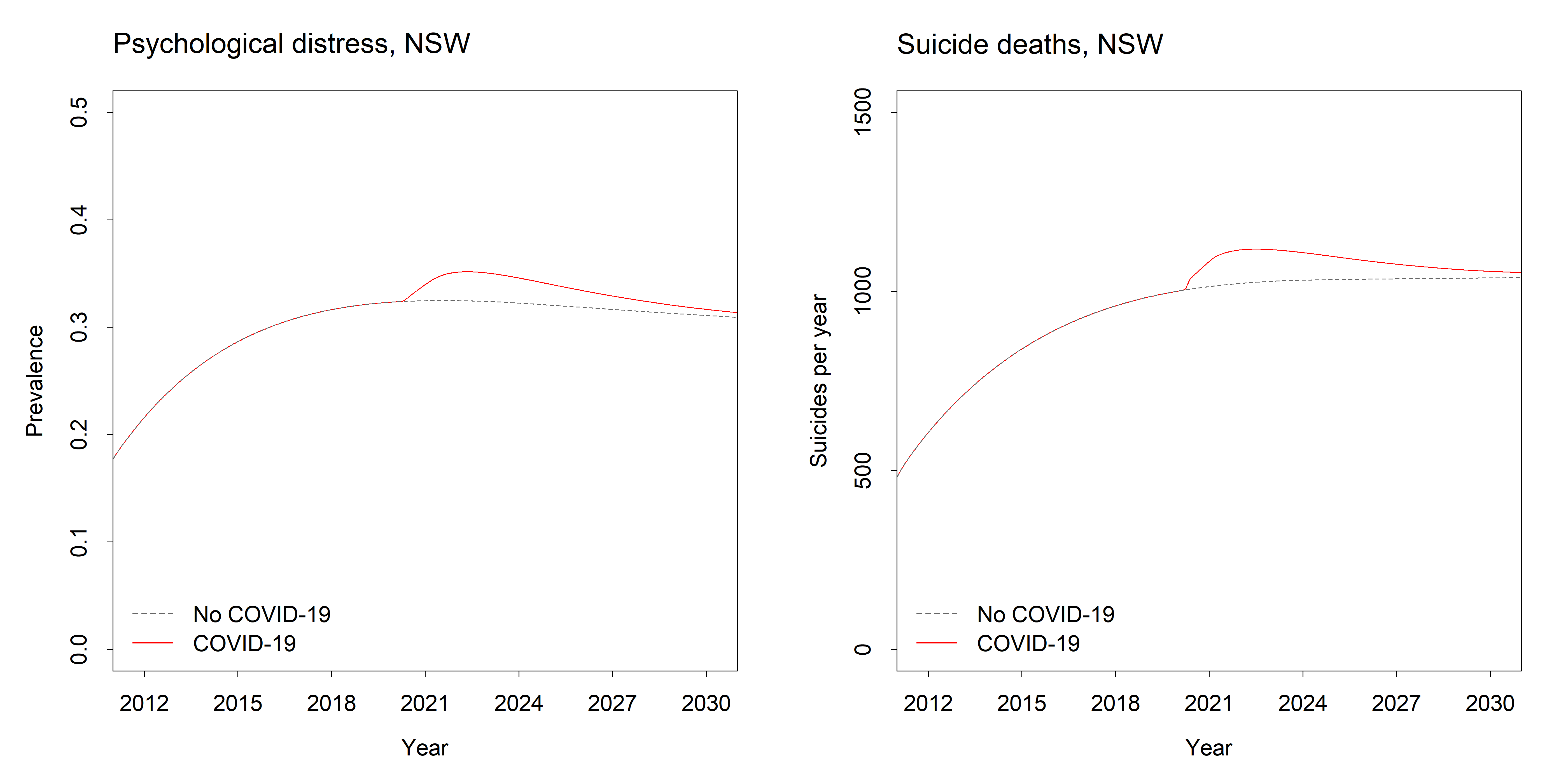


Figure S14. Modelled impact of the continuing COVID-19 pandemic on psychological distress and suicide mortality.

Table S4. Parameters controlling the modelled effect of the COVID-19 pandemic on the incidence of psychological distress.

| Parameter | Value | Notes |
| --- | --- | --- |
|  |  |  |
| COVID-19 effect decay rate | 1 | Fractional rate per year at which the effect of COVID-related social dislocation and unemployment decays after the pandemic ends (i.e., after the effect starting year plus the effect duration) |
| COVID-19 effect duration (years) | 1 | Duration in years of the direct effect of COVID-related social dislocation and job loss on psychological distress incidence. The default value (1) assumes that the direct effect of the COVID-19 pandemic persists for a period of 1 year. |
| COVID-19 effect starting year | 2020.167 | Default value (2020.167) assumes that significant social dislocation and job loss due to the COVID-19 pandemic commences in March 2020 |
| Maximum COVID-19 effect | 1.303974 | Maximum effect of COVID-related social dislocation and unemployment on psychological distress incidence. The default value (1.304) assumes a maximum increase in the per capita rate of psychological distress onset of 30.4%. |
| Years to reach maximum effect | 0.25 | Default value (0.25) assumes that the maximum effect of COVID-related social dislocation and unemployment on psychological distress incidence is reached in 3 months |
|  |  |  |

References

Atkinson, J., Song, Y. J. C., Merikangas, K. R., Skinner, A., Prodan, A., Iorfino, F., Freebairn, L., Rose, D., Ho, N., Crouse, J., Zipunnikov, V., Hickie, I. B., 2020. The science of complex systems is needed to ameliorate the impacts of COVID-19 on mental health. Front. Psychiatry 11, 606035.

Moreno, C., Wykes, T., Galderisi, S., Nordentoft, M., Crossley, N., Jones, N., Cannon, M., Correll, C. U., Byrne, L., Carr, S., Chen, E. Y. H., Gorwood, P., Johnson, S., Kärkkäinen, H., Krystal, J. H., Lee, J., Lieberman, J., López-Jaramillo, C., Männikkö, M., Phillips, M. R., Uchida, H., Vieta, E., Vita, A., Arango, C., 2020. How mental health care should change as a consequence of the COVID-19 pandemic. Lancet Psychiatry 7, 813−824.

Occhipinti, J, Ho, N., Skinner, A., Song, Y. C., Hickie, I. B., 2021. Closing the gender gap: supporting the mental health of Australian women during the COVID-19 era. Brain and Mind Centre, Sydney. Available at: https://www.sydney.edu.au/content/dam/corporate/documents/brain-and-mind-centre/youth-mental-health/ymh_closing_the_gender_gap_in_mh.pdf.


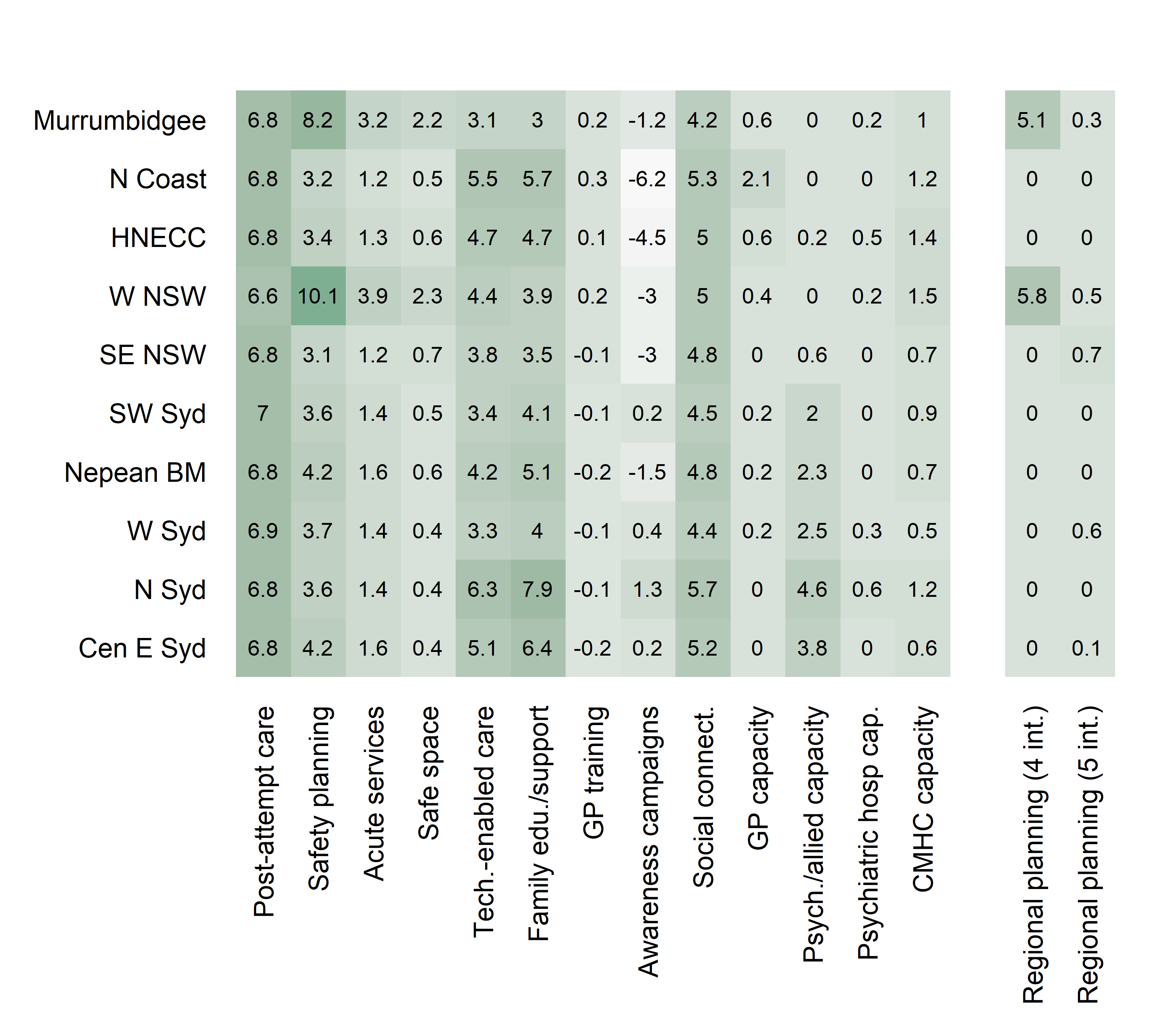


Figure S15. Percentage reductions in cumulative numbers of suicides over the period 2021−2031 projected under scenarios in which the 13 interventions in Supplementary Table S2 are implemented separately in each Primary Health Network (PHN) catchment (left panel). The panel on the right shows percentage reductions in projected numbers of suicides observed when the optimal combinations of four and five interventions for each PHN catchment are compared with the optimal state-level intervention combinations (combinations of interventions minimising state-level suicide mortality when implemented in all PHN catchments). Where the optimal combination of interventions at the PHN level is the same as the optimal state-level intervention combination, the percentage reduction in suicide mortality due to regional planning is (necessarily) equal to zero.
